# Understanding Contemporary Endometrial Cancer Survivorship Issues: Umbrella Review and Healthcare Professional Survey †

**DOI:** 10.3390/cancers17162696

**Published:** 2025-08-19

**Authors:** Tracey DiSipio, Jemma Turner, William Da Silva, Elizabeth Driscoll, Marta Preston, Krystel Tran, Nicla Varnier-Lui, Hui-Ling Yeoh, Dayajyot Kaur, Kathryn Alsop, Sandra C. Hayes, Monika Janda, Rosalind R. Spence

**Affiliations:** 1 School of Public Health, The University of Queensland, Herston, QLD 4006, Australia; 2 School of Medicine, The University of Notre Dame, Fremantle, WA 6160, Australia; 3 Australian Centre for Precision Health and Technology, Griffith University, Gold Coast, QLD 4222, Australia; 4 Medical School, The University of Queensland, Herston, QLD 4006, Australia; 5 Department of Emergency Medicine, Albany Medical Centre, Albany, NY 12208, USA; 6 The Kinghorn Cancer Centre, Darlinghurst, NSW 2010, Australia; 7 St Vincent’s Clinical School, University of New South Wales, Sydney, NSW 2033, Australia; 8 Department of Gynaecology Oncology, Chris O’Brien Lifehouse, Camperdown, NSW 2050, Australia; 9 Department of Radiation Oncology, Peter MacCallum Cancer Centre, Melbourne, VIC 3000, Australia; 10 The Sir Peter MacCallum Department of Oncology, University of Melbourne, Parkville, VIC 3010, Australia; 11 Department of Gynaecological Oncology, Westmead Hospital, Westmead, NSW 2145, Australia; 12 Peninsula Health, Frankston, VIC 3199, Australia; 13 Western Health, Footscray, VIC 3011, Australia; 14 Australia New Zealand Gynaecological Oncology Group (ANZGOG), Camperdown, NSW 2050, Australia; 15 Cancer Council Queensland, Fortitude Valley, QLD 4006, Australia; 16 Centre for Health Services Research, The University of Queensland, Woolloongabba, QLD 4102, Australia; 17 School of Health Science and Social Work, Griffith University, Nathan, QLD 4111, Australia

**Keywords:** endometrial cancer, patient-reported outcomes, survivorship, umbrella review

## Abstract

**Background/Objectives**: Advances in endometrial cancer treatment, such as minimally invasive surgery and the introduction of targeted therapies, could influence survivorship and result in changes in issues experienced by survivors of endometrial cancer. To improve endometrial cancer survivorship, it is first necessary to understand it. Therefore, the purpose of this study was to describe contemporary endometrial cancer survivorship issues. **Methods**: This study involved an umbrella review and cross-sectional survey of healthcare professionals. The umbrella review involved searching Medline (2013–2023) to identify published reviews describing survivorship outcomes in endometrial cancer survivors. Articles were screened and survivorship outcomes were extracted. Healthcare professionals working with patients diagnosed with endometrial cancer were asked to (1) rate the relevance of each survivorship outcome identified in the review, and (2) list any additional issues considered of clinical relevance. **Results**: Following the screening of 201 articles (159 papers excluded: *n* = 1 duplicate; *n* = 158 ineligible), 42 review articles were included and 25 survivorship outcomes identified including physical health (e.g., fatigue, pain), fertility (e.g., parity), quality of life (e.g., cognitive wellbeing), treatment-related toxicities, and mental health outcomes (e.g., anxiety). The cross-sectional survey was completed by 37 healthcare professionals (two-thirds being medical oncologists or gynaecological oncologists) working across Australia and New Zealand, with outcomes rated as relevant by 30% (for cachexia) to 100% (for body weight/obesity and functional wellbeing of respondents. An additional 28 survivorship issues not identified in the review were reported by healthcare professionals. **Conclusions**: The current endometrial cancer survivorship literature is limited. The review, together with expert input from healthcare professionals, identified 53 survivorship issues. These findings will now inform development of a survey to explore the frequency, impact, and severity of survivorship issues from the perspective of endometrial cancer survivors, the results of which will guide the provision of supportive cancer care, in addition to guiding research and clinical practice.

## Simple Summary

As the incidence of endometrial cancer rises and treatment options advance, the number of endometrial cancer survivors continues to increase. There is a need to understand the contemporary issues faced by survivors to inform healthcare services and improve survivorship. Therefore, an umbrella review and cross-sectional healthcare professional survey were conducted to describe contemporary issues. Collectively, 53 survivorship issues were identified (e.g., fatigue, parity, anxiety) that will now inform the development of a survey to explore contemporary survivorship issues from the perspective of endometrial cancer survivors and identify new or shifting priorities for survivorship care.

## 1. Introduction

Uterine cancer is the most prevalent of the gynaecological cancers and sixth most common female cancer globally [1]. There are two main types of uterine cancer—endometrial cancer, which originates in the epithelial lining of the uterus (endometrium) and accounts for 90% of uterine cancer cases; and uterine sarcomas, which develop in the muscle tissue and are a rarer form of uterine cancer [2]. The incidence of endometrial cancer is rising. Projections in the United States show a 55% increase in rates of endometrial cancer between 2010 and 2030 (from 27/100,000 women in 2010 to 42/100,000 women by 2030), with the aging population and increased incidence of obesity contributing to this rising incidence [3]. Endometrial cancer patients are typically diagnosed at an early stage; however, while the five-year survival rate is 80%, the number of deaths from endometrial cancer is also increasing primarily due to population aging and population growth [1,4].

A ‘cancer survivor’ is commonly recognised as a person who has been diagnosed with cancer, from the time of diagnosis throughout their life [5]. Endometrial cancer survivors may experience a diverse array of physical, psychosocial, emotional, and practical challenges that can begin before being diagnosed and persist through treatment and beyond [6]. Despite being a rapidly growing population, beyond conventional clinical endpoints, such as recurrence or death, little is known about the unique challenges faced by women following diagnosis [7]. The limited endometrial cancer survivorship research primarily precedes the introduction of treatment advancements that include minimally invasive surgical approaches, refinement of radiotherapy and chemotherapy protocols, and the introduction of immunotherapy and targeted therapies [7,8]. Differences in toxicity profiles between traditional and contemporary treatments will influence survivorship—potentially reducing the frequency and impact of some concerns, while concurrently introducing contemporary concerns and worsening the impact of others [7,9].

A comprehensive understanding of the issues faced by endometrial cancer survivors is necessary to inform the development of targeted healthcare initiatives and services to assist in a holistic recovery such as psychosocial support, rehabilitation and long-term monitoring. It is also integral to ensuring that services are tailored to the patients who most require them (including priority groups such as people from culturally and linguistically diverse backgrounds, older people, low-resource settings) and address the needs of greatest priority. Therefore, the overall objective of this research, which includes an umbrella review and clinician survey, was to develop an understanding of contemporary survivorship issues experienced by women with endometrial cancer, and to use this information to inform relevant supportive endometrial cancer care.

## 2. Materials and Methods

This project used a multi-stage approach to identify established and contemporary endometrial cancer survivorship issues. Stage 1 was an umbrella review that aimed to synthesise the existing evidence base on survivorship outcomes in endometrial cancer survivors. The research questions of this umbrella review were:

(i) What survivorship outcomes have been reported in endometrial cancer survivors; and
(ii) How have these survivorship outcomes been measured in research?

A preliminary search for previous umbrella reviews addressing this research question was conducted (Joanna Briggs Institute Evidence Synthesis, The Cochrane Library) and no results were found.

Stage 2 was a healthcare professional (HCP) survey that aimed to provide expert input on the contemporary survivorship issues observed in clinical practice. The research questions of this survey were:

(i) Do HCPs perceive each survivorship outcome identified from the umbrella review as being relevant for this population; and
(ii) Are there additional survivorship issues that HCPs view as being relevant that were not identified in the umbrella review?

### 2.1. Stage 1: Umbrella Review

The review followed the Joanna Briggs Institute (JBI) umbrella review method [10] and was registered with Open Science Framework (https://doi.org/10.17605/OSF.IO/KXWJQ).

#### 2.1.1. Inclusion Criteria

Types of participants: The population of interest were those diagnosed with endometrial cancer, and only reviews that presented results specific to endometrial cancer were included in the umbrella review.

Context/setting: The context and setting were not limited by any factors (e.g., cultural, geographical, health care settings).

Outcomes of interest: The primary outcomes of interest of this umbrella review were endometrial cancer survivorship outcomes. Survivorship outcomes, similar in definition to health-related outcomes [11], were defined as the health consequences brought about by a health condition, treatment of a health condition, or as a result of an interaction with the health care system. For example, possible survivorship outcomes include, but are not limited to, quality of life, physical health, mental health, and fertility outcomes. Survivorship outcomes reported at any time across the cancer continuum (i.e., from point of diagnosis through to end of life) [12] were eligible. Reviews were excluded if the only outcome reported was related to survival (e.g., overall survival, progression-free survival), which was not the intent of this umbrella review, or short-term surgical adverse events (e.g., length of hospital stay, intraoperative events), which are considered acute effects rather than survivorship issues.

Types of studies: All review types published in the past 10 years (2013–2023) were eligible for inclusion in this umbrella review (i.e., systematic review, meta-analysis, narrative review, descriptive review, scoping review, critical review, literature review, rapid review). Reviews published in English were included.

#### 2.1.2. Search Strategy

The search strategy was based on the PICOS framework [13] as follows: Population (people diagnosed with endometrial cancer), Outcome (survivorship outcomes), and Study (reviews). In this context, “intervention” and “comparison” elements were not applicable. One author (J.T.) completed an initial test search in consultation with a research librarian, then in collaboration with a senior author (R.S.) amended the strategy to ensure results were comprehensively identified. A search was conducted using MEDLINE in September 2023 (J.T.) to identify relevant papers published in the past 10 years (from January 2013) as this timeframe coincides with the integration of advancements in treatment in standard care. As per the JBI manual for evidence synthesis, MEDLINE was deemed sufficient to address the research questions of this umbrella review [10]. The search strategy included a combination of terms related to the population, outcomes, and study design (Supplementary Information S1). Records were managed in Microsoft Excel and Endnote X20 [14,15]. EndNote X20 was utilised to de-duplicate records.

#### 2.1.3. Screening and Selection

Two investigators (J.T., W.D.) independently screened titles and abstracts of articles that resulted from the initial database search. All articles that met the inclusion criteria were retrieved in full text. Full-text articles were screened (J.T., W.D.) using the hierarchy of criteria for exclusion (Supplementary Information S2) and discrepancies were resolved in discussion with senior authors (R.S., T.D.).

#### 2.1.4. Data Extraction

Data extraction was performed independently by the investigators (T.D., J.T., M.P., E.D., K.T., N.L., H.L.Y., D.K., W.D., R.S.) using a standardised data extraction spreadsheet and checked for accuracy (W.D., R.S., B.B.). All survivorship outcomes reported in the included reviews were extracted regardless of whether these outcomes were a priori defined outcomes of the review.

Key information extracted from each review included review characteristics (i.e., citation details, review type, review aim, number of studies with endometrial cancer survivors included, study designs, publication range of included studies), population characteristics (i.e., average age, time since diagnosis), outcomes (i.e., survivorship outcomes, measurement tools), quality appraisal (i.e., appraisal tools reported in the review, quality of the studies as reported in the review). Critical appraisal of each review was not performed as this would provide minimal value to the aim of this umbrella review (i.e., to collate a list of survivorship outcomes) and as the magnitude and direction of results were not intended to be synthesised. Instead, information on the quality of primary studies as reported within each review was extracted.

#### 2.1.5. Data Synthesis

Survivorship outcomes were grouped into categories based on clinically meaningful domains (e.g., mental health category includes outcomes related to depression, anxiety, and stress). A narrative synthesis of the findings was performed focusing on the objectives to describe (i) what endometrial cancer survivorship outcomes have been reported in research, and (ii) how these outcomes were measured. A list of all identified survivorship outcomes was then created for use in Stage 2 of this research.

### 2.2. Stage 2: Healthcare Professional Survey

#### 2.2.1. Study Design and Participants

Following ethical approval (Griffith University Human Research Ethics Committee (2024/200)), HCPs who have worked clinically with people diagnosed with endometrial cancer in Australia or New Zealand in the past five years were invited to complete an online, cross-sectional survey. The survey was designed to collect information about the relevance of the 34 survivorship outcomes identified via the umbrella review, from the perspective of the HCP. HCPs were asked to consider whether each outcome was of relevance when supporting people with endometrial cancer, taking into account how often the issues were encountered in practice, and how important and burdensome it was for the affected women. Responses were “yes”, “no”, or “unsure”. There was also a free-text field to list any additional survivorship issues believed to be relevant but not listed already in the survey. Our definition of survivorship issues used for the survey was broader than the definition of survivorship outcomes used in the umbrella review; survivorship issues holistically encompassed all issues or concerns that could influence any aspects of life following endometrial cancer diagnosis.

Information about participant characteristics, including the type of HCP and clinical experience (years since graduation, years working with people with endometrial cancer, and number of people with endometrial cancer seen per month) were also collected. The wording of survey questions was constructed by the research team that included clinicians, epidemiologists, and behavioural scientists. The survey was piloted by the clinicians in the team, with the intent to ensure accuracy and appropriateness of survivorship terms, as well as clarity and purpose of questions. The survey took approximately 10 min to complete (Supplementary Information S3).

#### 2.2.2. Data Collection

An online survey link was distributed to HCPs via the Australia New Zealand Gynaecological Oncology Group (ANZGOG) membership base. ANZGOG is the peak gynaecological cancer organisation in Australia and New Zealand, including clinical members representing medical doctors, gynaecological oncology nurses, and allied health professionals. The online survey was made available for nine weeks (April–June 2024) and distributed through email newsletters and at the ANZGOG Annual Scientific Meeting held in New Zealand (April 2024). Participation was voluntary and consent was obtained. All responses were anonymous. Data were collected using Research Electronic Data Capture (REDCap), a secure, web-based platform [16,17].

#### 2.2.3. Data Analysis

Descriptive statistics using frequencies and percentages were used to describe sample characteristics. Means and standard deviations were calculated to summarise normally distributed data, and median and ranges when data were not normally distributed. The proportion of positive responses for relevance of each item are presented. Free-text responses were reviewed for survivorship issues not already included in the survey list and new issues were placed into broad categories. Category names and grouping of survivorship issues within categories was determined by discussion and consensus among authors (T.D., R.S., K.T., E.D., N.L., K.A., S.H., M.J.), with representation of clinicians and researchers. Category names were chosen to clearly reflect the free-text responses and prioritizing clinically relevant terms that reflected language used in the reviewed literature.

## 3. Results

### 3.1. Stage 1: Umbrella Review

#### 3.1.1. Review Characteristics

**Figure 1:**
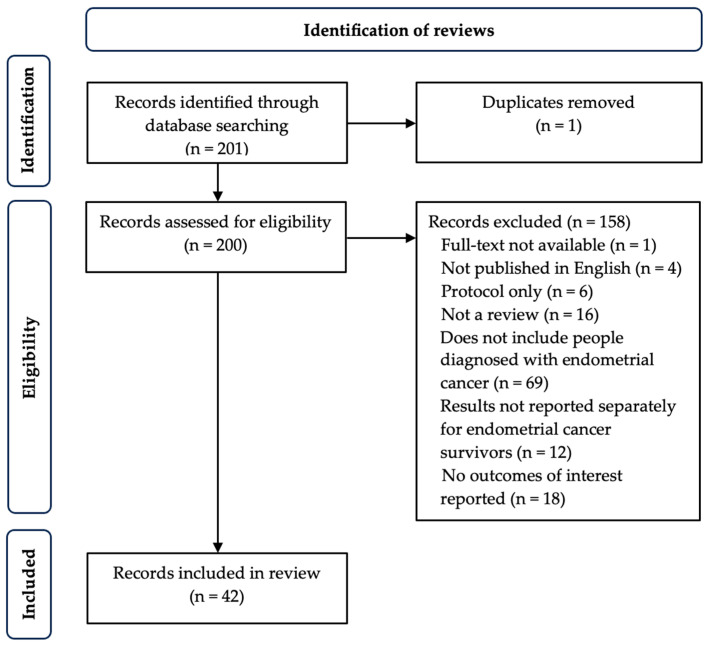
Flow chart of included studies.

A total of 201 reviews were identified and screened. After removal of one duplicate and 158 ineligible articles, 42 reviews reporting survivorship outcomes were included and data were extracted for synthesis (Figure 1).

The characteristics and outcomes of the 42 included reviews are presented in Supplementary Information S4. Twenty-five reviews were systematic reviews, with eight including a meta-analysis. The remaining 17 reviews were non-systematic, narrative, or literature reviews. Reviews included primary studies published between 1981 and 2021. Reviews included studies with participants ranging in age from 13 to 88 years and included women with diagnoses of stages I to IV including recurrent disease. Treatments were often mixed (i.e., more than one type of treatment per individual and/or a mixture of treatments within one review) and included surgery only, adjuvant chemotherapy, radiotherapy, hormonal therapy, and fertility-sparing treatment. Menopausal status was a characteristic of interest; however, it was only reported in four reviews (*n* = 2 reviews included studies among pre-menopausal women only [18,19]; *n* = 1 review included studies among post-menopausal women only [20]; *n* = 1 review included studies of pre- and post-menopausal women [21]). Similarly, time since diagnosis was poorly reported, and therefore the stage of the cancer continuum represented by the included reviews is unclear. There was extensive heterogeneity of study populations, timing of assessments, and tools used to assess outcomes in studies within and between reviews. Reviews described most primary studies as being of low quality (e.g., primarily retrospective designs with survivorship outcomes being considered secondary or exploratory outcomes).

#### 3.1.2. Survivorship Outcomes

Survivorship outcomes (*n* = 25) and the measurement instruments identified in the included reviews are listed in Table 1. Five main categories of outcomes were identified: physical health outcomes, fertility-related outcomes, quality of life, treatment-related toxicities, and mental health outcomes.

#### 3.1.3. Physical Health Outcomes

Eleven physical health outcomes, such as lymphoedema and sleep were described in the included reviews (Table 1). A range of measurement tools were reported for measuring physical health. Examples of variations include: (a) self-report or objective measures (e.g., lymphoedema was measured via a patient questionnaire, circumferential measurements of limbs, and/or medical record of patient complaint/clinical evaluation); (b) general or specific self-reported questionnaires (e.g., self-reported sleep quality as measured via one item of a general quality of life questionnaire (such as the EORTC QLQ-C30) [22,23,26,30], or sleep-specific questionnaire (such as the Pittsburgh Sleep Quality Index) [6,26]; and (c) numerous validated/reliable measures were available for some outcomes but limited for other outcomes (e.g., numerous tools were reported to measure fatigue, while there were limited tools to measure abdominal discomfort). A full list of measurement instruments for physical health outcomes are provided in Table 1.

#### 3.1.4. Fertility-Related Outcomes

Fertility-related outcomes, including gravidity and parity post-treatment, were reported by 20 reviews [18,19,20,21,33,34,42,43,44,45,46,47,48,49,50,51,52,53,54,55]. It was often unclear how information on pregnancy and live birth rates were collected (e.g., medical record extraction or self-report).

#### 3.1.5. Quality of Life

Quality of life was reported as an outcome in 11 reviews [6,22,23,24,26,30,35,37,41,56,57]. Our search found that most of the research used validated quality of life tools to measure global quality of life and/or domains. Examples of endometrial cancer-specific instruments include the European Organization for Research and Treatment of Cancer Quality of Life Questionnaire-Endometrial cancer module (EORTC QLQ-EN24) and the Functional Assessment of Cancer Therapy-Endometrial (FACT-En) (refer to Table 1 for a full list).

#### 3.1.6. Treatment-Related Toxicities

Treatment-related toxicities were reported in six reviews [20,22,27,28,29,58]. There was minimal detail on how treatment-related toxicities were measured (i.e., self-report or medical records). The Functional Assessment of Chronic Illness Therapy-Fatigue (FACIT-Fatigue) was mentioned in measuring fatigue as a treatment-related toxicity [29], and one review [22] reported multiple tools (Late Effects Normal Tissues-Subjective, Objective, Management Analytic [LENT-SOMA]; Radiation Therapy Oncology Group/European Organization for Research and Treatment of Cancer late toxicity scale; Common Terminology Criteria for Adverse Events [CTCAE]).

#### 3.1.7. Mental Health Outcomes

Mental health issues were addressed in two systematic reviews [6,26]. Anxiety, depression, and distress/stress were primarily measured using validated tools, including the Beck Depression Inventory, Brief Symptom Inventory-18, Hospital Anxiety and Depression Scale, Quality of Life in Adult Cancer Survivors, and Structured Interview Guide for the Hamilton Anxiety/Depression Scales (refer to Table 1 for a full list).

### 3.2. Stage 2: Healthcare Professional Survey

A total of 37 HCPs completed the survey with two-thirds being medical oncologists or gynaecological oncologists. HCP reported having worked with endometrial cancer patients for an average of 11 years (range 2, 28) and seeing an average of five endometrial cancer patients per month (range < 1, 30) (Table 2). Seventy percent reported being ten or more years post-graduation.

The proportions of HCPs agreeing that a survivorship outcome, as identified from the reviews, was relevant for supporting people with endometrial cancer are presented in Table 3 with additional issues identified by HCPs listed in Table 4. The proportion of HCPs who rated an outcome as relevant ranged from 29.7% (for cachexia) to 100% (for body weight/obesity and functional wellbeing). Of the outcomes presented, 19 were identified as relevant by >70% of participants.

HCPs identified an additional 28 survivorship issues not extracted from the reviews (Table 4). Five new categories were identified: sexual wellbeing, work and financial outcomes, return to usual activities, surveillance and recurrence (noting that survival outcomes were excluded from the umbrella review), and communication and access to information. While sexual wellbeing is a new category, there were previously two related outcomes captured under the quality of life (outcome: sexual wellbeing) and physical health (outcome: sexual function) categories. The addition of two new issues related to sexual wellbeing prompted the creation of the new category. Nomenclature modifications were made to two categories that had emerged from the umbrella review: (i) mental health, psychological and emotional outcomes (previously “mental health outcomes”), and (ii) treatment-related toxicities and health outcomes (previously “treatment-related toxicities”). No meaningful grouping was determined for two of the new issues: failure of radiation and living with late stage/metastatic disease; these issues are listed under the category “other”.

## 4. Discussion

Endometrial cancer survivorship is an evolving topic of research, particularly in light of uncertainty for how new treatments, such as immunotherapy, will impact long-term survivorship. This review identified 25 survivorship outcomes, with the majority of these rated as relevant by >70% of HCPs (all survivorship outcomes except for cachexia, sarcopenia, parity and gravidity), based on how frequently the outcome arises in clinical practice and how important or burdensome the outcomes are for affected women. These results suggest that the survivorship outcomes explored in the literature remain relevant. However, HCPs identified a further 28 issues, in addition to the outcomes identified from the review, with the combined issues representing 11 categories: quality of life, fertility-related outcomes, mental health, psychological and emotional outcomes, physical health, treatment-related toxicities and health outcomes, sexual wellbeing, work and financial outcomes, return to usual activities, surveillance and recurrence, communication and access to information, and other (i.e., failure of radiation, living with late stage/metastatic disease). These findings will now be used to inform Stage 3 of this project: the development of a consumer survey to explore the frequency, impact and severity of survivorship issues as reported by patients diagnosed with endometrial cancer.

The survivorship issues identified here are like survivorship concerns described in the broader cancer literature such as quality of life, financial toxicity, fear of recurrence, and fertility [59]. However, it is unclear how well these issues reflect the contemporary issues being experienced because of the evolution of treatment [8]. Results of recent clinical trials (e.g., PORTEC-2 and PORTEC-3) [60,61]) have led to more tailored approaches to adjuvant treatments. These achieved minimisations of toxicities and equivalent outcomes and led to updated evidence-based guidelines including the introduction of combined chemoradiation with improvement in survival and the development of a trial investigating the role of immunotherapy in the adjuvant setting [7,8]. In many cases, the acute toxicity profiles are improved in these contemporary treatment protocols, but the impact on survivorship issues is rarely considered during the clinical trials [62]. To ensure that improvements in survival concurrently come with improvements in quality of survival, there is a clear need to understand how contemporary advancements in medical treatment influence survivorship. This move is exemplified in the RAINBO clinical trial program investigating de-escalation of treatment in the context of a favourable molecular profile [63]. Secondary outcomes are predetermined to include toxicity and quality of life analyses which will yield this critical information and demonstrates the increased interest in survivorship outcomes.

The results of the HCP survey provide further insight into the survivorship issues in current clinical practice. The additional categories of survivorship issues identified in the survey, such as work and financial outcomes, and return to usual activities, may be associated with an increasing incidence among younger women who, when compared with older women, may require a more full and faster return to work following treatment, and who will also likely need to remain in the workforce for longer. US SEER data show that while age-adjusted incidence rates between 2003 and 2021 increased among all age groups, the largest increase was seen among those aged <50 years [64], suggesting the need to address survivorship concerns across a wide range of ages and life stages. The added categories of surveillance and recurrence, and communication and access to information, may be of growing importance as treatment options increase in number and complexity. For example, the increasing application of fertility-sparing treatment (influenced both by the increasing numbers of younger women being diagnosed, and the development of improved surgical techniques), and emerging treatment approaches informed by molecular prognostic grouping, lead to more complex treatment decisions and a greater need for surveillance and access to information [65]. The umbrella review and HCP survey have provided insight into the established and contemporary endometrial cancer survivorship issues. However, to advance the provision of survivorship care in endometrial cancer, there is now an imperative to develop a better understanding of the priority issues for those living with and beyond endometrial cancer.

### Strengths and Limitations

The results of the umbrella review were influenced by the limitations of the included reviews. The survivorship outcomes identified from our umbrella review reflect topics that were of interest to conduct a review or when enough original research was available to warrant a review, and likely for topics where measurement tools are available. Newer treatments, such as combined chemoradiation, or anti-PD1-based immune therapy, may not be well represented in these results and likely reflect that it is too early for the accumulation of evidence required to prompt or enable a review. Nonetheless, acknowledgement of this limitation, at least in part, led us to supplement the umbrella review findings with the cross-sectional survey. The matter of which issues are most important to survivors, based on frequency, magnitude and impact, was not addressed by this research. Further exploration is needed to ensure the consumer view is reflected when describing the survivorship issues faced after endometrial cancer diagnosis.

Our umbrella review has some limitations. This review excluded non-English publications thereby omitting potentially relevant reviews and limiting the diversity of populations included. This review did not explore issues among caregivers of people diagnosed with endometrial cancer, and who may also be impacted by survivorship issues and require support. Therefore, experiences among other diverse population groups and caregivers warrant separate review. Finally, the review search strategy used terms related to health consequences of survivorship and did not capture all relevant issues, such as financial toxicity. However, the HCP survey provided the extended scope to ensure these issues were captured.

The HCP survey intentionally targeted clinicians in Australia and New Zealand. However, this may limit the generalisability of the results, with survivorship issues likely influenced by current clinical practice in the geographical region, as well as non-clinical factors such as cultural practices or available survivorship support. Potentially more outcomes would have been identified if there were more HCP participants. However, the targeted recruitment strategy, via the ANZGOG mailing list and national conference, likely led to the recruitment of HCPs with relevant clinical experience and expertise, as well as an interest in gynaecological care and research. This is reflected in the characteristics of those who completed the survey; 700 graduated more than 10 years ago and have a median of 11 years working with people with endometrial cancer. A range of clinical areas are represented by the survey participants, including surgical, medical, and radiation oncology and allied health, providing a range of perspectives and understandings of the patient experience. It is possible that more in-depth data collection methods, such as focus groups, or a larger sample, would have led to identification of additional survivorship issues. However, it is expected that the 53 survivorship issues identified will be sufficient in quantity and variety to prompt comprehensive input from consumers in Stage 3 of this project.

## 5. Conclusions

There are more survivors than ever before living with the impacts of endometrial cancer diagnosis and treatment and the subsequent complex needs of survivorship. In addition to the burden of the disease to individuals, there is a significant public health cost of treatment and survivorship. To better manage survivorship concerns and understand what services are needed and where, there is a clear need for improved understanding of what issues are a priority and for whom. Importantly, there is a need to capture the needs of diverse populations, especially priority groups who experience health disparities.

The next step is to use these results to guide a survey of endometrial cancer survivors to explore consumer insights into the frequency, impact and severity of the identified survivorship issues. This will also allow for the identification of additional survivorship issues that have not been identified by review of the literature or expert input, ensuring that the needs of those living with and beyond endometrial cancer can be addressed.

## Figures and Tables

**Table 1:** Survivorship outcomes identified from the umbrella review.

Category	Outcome	Measurement Instruments
Physical health outcomes		
	Abdominal discomfort [20,22,23,24]	EORTC-QLQ-C30; SF-36
	Cachexia [25]	Weight loss; BMI; Biochemical abnormalities
	Fatigue [6,26,27,28,29]	BFI; EORTC-QLQ-C30; FACIT; FACIT-F; FAS; QLACS [fatigue domain]; SF-36
	Lymphoedema [30,31,32]	CaSUN; Circumferential measurements; GCLQ-K; MRI; Ultrasound; Validated lymphoedema questionnaire
	Obesity [19,21,26,33,34,35,36]	BMI
	Pain [6,24,26,28,37,38]	BPI; EORTC-QLQ-C30; Numerical rating scale (0–10); Perioperative clinical data; QLACS; SF-36
	Pelvic floor function [23,35,39]	APFQ; Motor evoked potential of sacral nerve; PFDI; PFM strength on digital palpation; PISQ-12
	Sarcopenia [40]	Average muscle radiation attenuation; Muscle mass measurement; Skeletal muscle index
	Sexual function [6,22,23,26,35,37,39,41]	ACOG Sexual Dysfunction Checklist; CALGB Sexual Functioning; FSFI; PROMIS; SAQ
	Sleep [6,26]	Actigraphy wristwatch + sleep log; EORTC-QLQ-C30; PSQI
	Urinary function [22,23]	APFQ; IIQ-SF; ISI; QUID; UDI-SF
Fertility-related outcomes		
	Gravidity [18,19,20,21,33,34,42,43,44,45,46,47,48,49,50,51,52,53]	Pregnancy rates
	Parity [18,20,21,33,34,42,43,44,45,46,47,48,51,52,53,54,55]	Live birth rates
Quality of life		
	Global quality of life [22,23,24,26,30,56,57]	EORTC-QLQ-C30; EORTC-QLQ-EN24; EQ-5D; FACT; FACT-En; FACT-G; Kupperman index; QOL-CS; QoR-40; RAND-36; RI10; SF-36; WHOQOL-BREF
	Cognition [6]	
	Emotional wellbeing [56,57]	
	Functional wellbeing [22]	
	Physical wellbeing [22,26,30,56]	
	Role functioning [22]	
	Sexual wellbeing [22,23,35,41,57]	
	Social wellbeing [22,56,57]	
Treatment-related toxicities		
	Treatment-related toxicities [20,22,27,28,29,58]	CTCAE; EORTC; FACIT; LENT-SOMA; RTOG/EORTC late scoring scheme
Mental health outcomes		
	Anxiety [6,26]	BDI; HADS; IDAS; PSS; SF-36; SIGH-AD
	Depression [6,26]	BDI; HADS; IDAS; SF-36; SIGH-AD
	Distress/stress [6]	BSI-18; QLACS

Abbreviations: ACOG: American College of Obstetrics and Gynecologists; APFQ: Australian Pelvic Floor Questionnaire; BDI: Beck Depression Inventory; BFI: Brief fatigue inventory; BMI: Body Mass Index; BPI: Brief Pain Inventory; BSI-18: Brief Symptom Inventory-18; CALGB: Cancer and Leukemia Group-B; CaSUN: Cancer Survivors’ Unmet Needs Measure; CTCAE: Common Terminology Criteria for Adverse Events; EORTC: European Organization for Research and Treatment of Cancer; EORTC QLQ-C30: European Organisation for Research and Treatment Core Quality of Life Questionnaire—Core 30; EORTC QLQ-EN24: European Organisation for Research and Treatment Quality of Life Questionnaire Endometrial cancer module; EQ-5D: EuroQol-5 Dimension; FACIT: Functional Assessment of Chronic Illness Therapy; FACIT-F: Functional Assessment of Chronic Illness Therapy—Fatigue; FACT: Functional Assessment of Cancer Therapy; FACT-En: Functional Assessment of Cancer Therapy—Endometrial; FACT-G: Functional Assessment of Cancer Therapy—General; FAS: Fatigue Assessment Scale; FSFI: Female Sexual Function Index; GCLQ-K: Gynecologic Cancer Lymphedema Questionnaire (Korean version); HADS: Hospital Anxiety and Depression Scale; IDAS: Inventory of Depression and Anxiety Symptoms; IIQ-SF: Incontinence Impact Questionnaire- Short Form; ISI: Insomnia Severity Index; LENT-SOMA: Late Effects Normal Tissues-Subjective, Objective, Management Analytic; MRI: Medical Resonance Imaging; PFDI: Pelvic Floor Dysfunction Inventory; PFM: Pelvic floor muscle; PISQ-12: Pelvic Organ Prolapse/Urinary Incontinence Sexual Questionnaire; PISQ-IR: Pelvic Organ Prolapse/Urinary Incontinence Sexual Questionnaire- IUGA Revised; PSQI: Pittsburgh Sleep Quality Index; PROMIS: Patient Reported Outcome Measurement System; PSS: Perceived Stress Scale; QLACS: Quality of Life in Adult Cancer Survivors; QOL-CS: Quality of Life-Cancer Survivors; QoR-40: Quality of Recovery-40 Scale; QUID: Questionnaire for Urinary Incontinence Diagnosis; RAND-36: RAND 36-item Health Survey; RI10: Recovery Index-10; RTOG: Radiation Therapy Oncology Group; SABIS-G: sexual adjustment and body image scale-gynecologic cancer; SAQ: Sexual Activity Questionnaire; SF-12: 12-item Short Form Health Survey; SF-36: 36-item Short Form Health Survey; SIGH-AD: Structured Interview Guide for the Hamilton Anxiety/Depression Scales; UDI-SF: Urinary Distress Inventory-Short Form; WHOQOL-BREF: World Health Organization’s Quality of Life instrument-abbreviated version.

**Table 2:** Healthcare professional participant characteristics (N = 37).

Characteristics	*n*	%
Country of health care provision		
Australia	31	(83.7)
New Zealand	6	(16.2)
Type of healthcare professional		
Medical oncologist	12	(32.4)
Gynaecological oncologist	11	(29.7)
Oncology nurse	8	(21.6)
Nurse	3	(8.1)
Radiation oncologist	1	(2.7)
Psychologist	1	(2.7)
Oncology research clinical trials	1	(2.7)
Years of clinical experience		
Graduated < 10 years ago	9	(24.3)
Graduated 10+ years ago	26	(70.3)
Missing	2	(5.4)
	**median**	**(minimum, maximum)**
Number of years working with endometrial cancer patients	11.0	(2, 28)
Approximate number of endometrial cancer patients seen per month	5.0	(<1, 30)

**Table 3:** Healthcare professional ratings of survivorship outcomes.

Outcome	Relevant, %
**Physical health outcomes**	
Abdominal discomfort	78.4
Body weight/obesity	100.0
Cachexia	29.7
Fatigue	97.3
Lymphoedema	73.0
Pain	83.8
Pelvic floor function	89.2
Sarcopenia	48.6
Sexual function	89.2
Sleep quality	91.9
Urinary function	83.8
**Fertility-related outcomes**	
Live birth rate (parity)	40.5
Pregnancy rate (gravidity)	40.5
**Quality of life**	
Cognitive wellbeing	83.8
Emotional wellbeing	97.3
Functional wellbeing	100.0
Physical wellbeing	97.3
Sexual wellbeing	97.3
Social wellbeing	94.6
Role functioning ^a^	-
**Treatment-related toxicities**	
Treatment-related toxicities	78.4
**Mental health outcomes**	
Anxiety	94.6
Cognition	75.7
Depression	94.6
Distress/Stress ^a^	-

^a^ These items were not included in the survey. Categories shown in bold text.

**Table 4:** Additional survivorship outcomes identified by healthcare professionals.

**Sexual wellbeing** Dilator use Sexual function vs. libido **Mental health, psychological and emotional outcomes** Fear of recurrence Interpersonal relationships particularly partner relationship Social support Loneliness **Physical health outcomes** Cardiovascular function/general fitness/physical fitness Weight loss **Treatment-related toxicities and health outcomes** Immune adverse event survivorship Bone health and pelvic insufficiency fractures Scarring/surgical scar tissue Risk of toxicity (premature) Menopause management ^1^ Complementary and alternative treatments ^2^ **Work and financial outcomes** Financial toxicity Returning to work **Return to usual activities** Ability to continue to undertake caring roles Changes to identity/roles and life values/goals Preservation of independence **Surveillance and recurrence** Risk of recurrence Imaging surveillance Treatment options for recurrence **Communication and access to information** Unmet information needs by patient Unmet information needs by family Peer advocacy Role of immunotherapy Complementary and alternative treatments ^2^ **Other** Failure of radiation Living with late stage/metastatic disease

^1^ Authors added “premature” to clarify assumed meaning of free text in survey. ^2^ Complementary and alternative treatment has been listed under two categories. Categories show in bold text.

## Data Availability

The data underlying this article are available in the article and in its online Supplementary Information.

## Supplementary Materials

The following supporting information can be downloaded at: https://www.mdpi.com/article/10.3390/cancers17162696/s1, S1: Search strategy; S2: Hierarchy of exclusion; S3: Health care professional survey; S4: Characteristics and outcomes of included reviews.

## Abbreviations

The following abbreviations are used in this manuscript:

HCP	Healthcare professional
JBI	Joanna Briggs Institute
ANZGOG	Australia New Zealand Gynaecological Oncology Group
REDcap	Research Electronic Data Capture
EORTC QLQ-EN24	European Organization for Research and Treatment of Cancer Quality of Life Questionnaire-Endometrial cancer module
FACT-En	Functional Assessment of Cancer Therapy-Endometrial
FACIT-Fatigue	Functional Assessment of Chronic Illness Therapy-Fatigue
LENT-SOMA	Late Effects Normal Tissues-Subjective, Objective, Management Analytic
CTCAE	Common Terminology Criteria for Adverse Events
