# Supplementary material for: Understanding Contemporary Endometrial Cancer Survivorship Issues: Umbrella Review and Healthcare Professional Survey
Source: Cancers (Basel). 2025 Aug 19;17(16):2696. doi: 10.3390/cancers17162696 (PMC12384384; doi:10.3390/cancers17162696)
Supplement: Supplementary file 1 [file cancers-17-02696-s001.zip › cancers-3761631-supplementary.pdf]

Supplementary Information S1. Search strategy

1. Endometrial cancer[TiAb] OR Uterine cancer[TiAb] OR Endometrial neoplasms[MeSH] OR Uterine neoplasms[MeSH]

AND

2. Treatment[TiAb] OR Surgery[TiAb] OR Chemotherapy[TiAb] OR Hormone Therapy[TiAb] OR Brachytherapy[TiAb] OR External Beam[TiAb] OR Immunotherapy[TiAb] OR Radiotherapy[MeSH] OR Radiation therapy[MeSH]

AND

3. Survivorship[TiAb] OR Health-related outcomes[TiAb] OR health-related concerns[TiAb] OR patient-reported outcomes[TiAb] OR PRO[TiAb] OR Patient reported concerns[TiAb] OR fatigue[TiAb] OR quality of life[TiAb] OR QoL [TiAb] OR sexual function[TiAb] OR Pelvic floor dysfunction [TiAb] OR Cognition[TiAb] OR Physical function[TiAb] OR Physical wellbeing[TiAb] OR Bone health[TiAb] OR Body composition[TiAb] OR Fertility[TiAb] OR Depression[TiAb] OR Anxiety[TiAb] OR Mental health[TiAb] OR Morbidity[TiAb] OR Mortality[TiAb] OR Bladder function[TiAb] OR Bowel function[TiAb] OR Outcome Assessment, Health Care[MeSH]

AND

4. Review[TiAb] OR Systematic Review[TiAb]

Supplementary Information S2. Hierarchy of exclusion

1	Publication	Not published in the past 10 years (2013-2023).
2	Publication	Full-text not available.
3	Publication	Not published in English.
4	Publication	Protocol only.
5	Publication	Not a review.
6	Population	Does not include people diagnosed with endometrial cancer.
7	Population	Results not reported separately for endometrial cancer survivors.
8	Outcome	No survivorship outcomes of interest reported.
9	Outcome	Survival outcomes only reported.
10	Outcome	Acute surgical adverse events only reported.

Supplementary Information S3. Health Care Professional Survey

Title: Contemporary survivorship issues in people with endometrial cancer:

a health professional survey

Do you consent to take part in this study?

Yes *(continue)*

No *(end of survey)*

Eligibility

Have you provided health care to women with endometrial cancer (from diagnosis to end of life) in the past 5 years, in Australia or New Zealand? *(tick)*

Yes - *continue*

No – *not eligible*

Respondent characteristics

Where have you provided healthcare to women with endometrial cancer in the past 5 years? *(tick)*

Australia

New Zealand

Both Australia and New Zealand

Type of health care professional: *(tick)*

Gynaecological surgeon / oncologist

Medical oncologist

Radiation oncologist

Oncology nurse

Palliative care nurse

Nurse

General Practitioner

Allied health professional (please specify below)

Other (please specify below)

If Allied health professional or other: *(describe)* …

Describe your clinical experience: *(tick)*

Graduated 0-2 years ago

Graduated 3-5 years ago

Graduated 6-10 years ago

Graduated 10+ years ago

How many years have you been working with people diagnosed with endometrial cancer?

###

Approximately how many people with endometrial cancer do you see per month?

###

Endometrial cancer survivorship issues

We are interested in understanding the contemporary survivorship issues experienced by people with endometrial cancer. Below is a list of survivorship issues that have been identified in the literature, please indicate the relevance of each issue, taking into account how often you encounter it in practice, and how important and burdensome it is for the affected women.

For each survivorship issue listed please:

1) Mark whether you believe this issue is relevant when supporting this population (Yes, No, Unsure)

Survivorship issue	Relevant?
**Quality of life**	Y / N / Unsure
Physical wellbeing	Y / N / Unsure
Social wellbeing	Y / N / Unsure
Functional wellbeing	Y / N / Unsure
Emotional wellbeing	Y / N / Unsure
Sexual wellbeing	Y / N / Unsure
Cognitive functioning	Y / N / Unsure
**Fertility-related outcomes**	Y / N / Unsure
Conception methods	Y / N / Unsure
Pregnancy rate	Y / N / Unsure
Pregnancy loss (e.g., miscarriage, termination, ectopic pregnancy)	Y / N / Unsure
Pregnancy complications (e.g., preterm delivery)	Y / N / Unsure
Live birth rate	Y / N / Unsure
**Mental Health Outcomes**	Y / N / Unsure
Anxiety	Y / N / Unsure
Depression	Y / N / Unsure

Cognition	Y / N / Unsure
**Physical Health Outcomes**	Y / N / Unsure
Body weight / Obesity	Y / N / Unsure
Sexual dysfunction	Y / N / Unsure
Abdominal discomfort	Y / N / Unsure
Pain	Y / N / Unsure
Pelvic floor function	Y / N / Unsure
Urinary function (e.g., urinary incontinence, urinary frequency)	Y / N / Unsure
Neurotoxicity	Y / N / Unsure
Fatigue	Y / N / Unsure
Sleep quality	Y / N / Unsure
Lymphoedema	Y / N / Unsure
Cachexia	Y / N / Unsure
Sarcopenia	Y / N / Unsure
**Adverse events / effects**	Y / N / Unsure
Clinical concerns (e.g., anemia, leukopenia, neutropenia, thrombocytopenia, hypertension, hypothyroidism, mucositis, rash)	Y / N / Unsure
Functional adverse events (e.g., pain, dyspnea, nausea, fatigue, diarrhoea, incontinence, fecal leakage)	Y / N / Unsure
Treatment-related toxicities	Y / N / Unsure

Please list any additional survivorship issues you believe are relevant, that have not been listed above:

1)

2)

3)

4)

5)

Supplementary Information S4: Characteristics and outcomes of included reviews

Physical health: ABDOMINAL DISCOMFORT						
**Author (year)**	**Study aim**	**Characteristics of primary studies[a]**	**Characteristics of participants**	**Survivorship outcomes reported[#]**	**Measurement instruments[a]**	**Quality appraisal as reported by the review**
Brennen et al (2020)[24]<br><br>Systematic review	Identify, evaluate and synthesize the evidence from the studies investigating the effect of non-surgical, non-pharmacological, pelvic floor muscle interventions on any type of pelvic floor dysfunction or health related quality of life in patients following any type of gynaecological cancer treatment.	N=4<br>3 randomised controlled trial<br>1 retrospective<br>Publication range: 1999-2014	N=134 endometrial cancer<br>Mean age: 60 years<br>Age range: 28-84 years<br>Cancer type: stage I-III<br>Treatment: surgery only, adjuvant chemotherapy, radiotherapy, mixed	Abdominal discomfort<br>Pelvic floor function[#]<br>Sexual function<br>Urinary function<br>Quality of life[#]	APFQ<br>CTCAE v.4<br>EORTC QLQ-C30<br>EORTC QLQ-CX24<br>IIQ-SF<br>ISI<br>Motor evoked potential of sacral nerve<br>PFM strength on digital palpation<br>PGI-I<br>QUID<br>SHF<br>SKQ<br>UDI-SF	PEDro Scale (randomised controlled trials): mean score 5/10<br><br>Cochrane risk of bias domains (randomised controlled trials): unclear risk of bias<br><br>NOS (non-randomised studies): mean score 6/9
Burke et al (2014)[44]<br><br>Literature review and practice recommendations	Review the risks and benefits of current treatment options and optimise treatment for women with endometrial cancer through creating evidence-based practice recommendations for diagnosis and treatment.	N=NR	N=NR<br>Treatment: adjuvant chemotherapy, radiotherapy, fertility-sparing treatment	Abdominal discomfort<br>Pain<br>Quality of life[#]	NR	NR
Mirabeau-Beale et al (2014)[22]<br><br>Literature review	Summarise the literature on quality of life for patients treated with definitive radiation for gynaecological	N=8<br>3 randomised controlled trial<br>1 prospective	N=NR<br>Treatment type: surgery only, adjuvant chemotherapy, radiotherapy, mixed	Abdominal discomfort<br>Sexual function<br>Urinary function<br>Quality of life[#]	ACOG Sexual Dysfunction Checklist	NR

	cancer, with a specific focus on patient reported outcomes.	1 retrospective<br>Publication range: NR		Treatment-related toxicities	CALGB Sexual Functioning<br>CTCAE<br>EORTC<br>EORTC-CX24<br>EORTC- EN24<br>EORTC QLQ-C30<br>EQ-5D<br>FACT<br>FACT-G<br>FSFI<br>GSCQ<br>HADS<br>LENT-SOMA<br>PROMIS<br>QOL-CS<br>RTOG/EORTC late scoring scheme<br>SAQ<br>SF-36<br>WHOQOL-BREF	
Prodromidou et al (2021)[20]<br><br>Systematic review and meta-analysis	Evaluate the effect of metformin and progesterone monotherapy on preserving fertility in endometrial cancer patients and examine the potential preventive role of metformin in breast cancer survivors and obese patients.	N=2<br>2 randomised controlled trial<br>Publication range: 2020	N=205 endometrial cancer<br>Mean age: 44 years<br>Cancer type: primary diagnosis, recurrent<br>Treatment: adjuvant chemotherapy, fertility-sparing treatment<br>Menopausal status: post-menopausal (n=32)	Abdominal pain<br>Gravidity<br>Parity<br>Treatment-related toxicities	Pregnancy rates<br>Live birth rates	Cochrane Risk of Bias tool: low risk of bias
**Physical health: CACHEXIA**						
**Author (year)**	**Study aim**	**Characteristics of primary studies[a]**	**Characteristics of participants**	**Health-related outcomes reported[#]**	**Measurement instruments[a]**	**Quality appraisal as reported by the review**

Alanazi et al (2021)[23]<br><br>Systematic review	Describe the sleep patterns among uterine cancer survivors and verify psychological and physical factors affecting their general quality of life.	N=10<br>4 randomised controlled trial<br>2 prospective<br>1 retrospective<br>2 cross-sectional<br>1 non-randomised controlled trial<br>Publication range: 2015-2019	N=1535 endometrial cancer<br>Cancer type: stage I<br>Treatment: surgery only, radiotherapy, mixed	Fatigue<br>Obesity<br>Pain<br>Sexual function<br>Sleep[#]<br>Quality of life[#]<br>Anxiety<br>Depression	Actigraphy wristwatch + sleep log<br>BMI<br>BSI-18<br>EORTC QLQ-C30<br>FACT-En<br>PSQI<br>PSS<br>QLACS<br>QOL-CS<br>SF-36	Melnyk's evidence pyramid
Anker et al (2019)[59]<br><br>Systematic review	Estimate the number of patients in the USA and EU with cachexia diagnosed with a common cancer type or other specific cancer types where cachexia is known to be a frequent.	N=4<br>4 observational<br>Publication range: 1981-2013	N=1280<br>Age range: 30-80 years<br>Cancer type: stage III/IV 45-64%, metastatic 57-61%	Cachexia	Measurement tool NR<br>Cachexia diagnosis: Weight loss ≥5% OR BMI <20 or <18.5 often + weight loss 2-5% or biochemical abnormalities	NR
**Physical health: FATIGUE**						
**Author (year)**	**Study aim**	**Characteristics of primary studies[a]**	**Characteristics of participants**	**Health-related outcomes reported[#]**	**Measurement instruments[a]**	**Quality appraisal as reported by the review**
Charo et al (2019)[51]<br><br>Literature review	Highlight recent advances in endometrial cancer research, focusing on surgical staging, sentinel lymph node mapping, adjuvant treatment, combination therapy, molecular biology and immunotherapy.	N=7<br>Phase III clinical trials<br>Publication range: 2015-2019	N=3923<br>Cancer type: primary diagnosis, recurrent, stage I-IV<br>Treatment: adjuvant chemotherapy, radiotherapy, mixed	Fatigue<br>Treatment-related toxicities	FACIT	NR

Chen et al (2021)[50]<br><br>Systematic review and meta-analysis	Evaluate the efficacy and safety of bevacizumab-combined chemotherapy in advanced/recurrent endometrial cancer.	N=7<br>2 randomised controlled trial<br>5 single-arm phase II trial<br>Publication range: 2007-2019	N=622<br>Average/median age: 62 years<br>Age range: 57-63 years<br>Cancer type: primary diagnosis, recurrent, stage III-IV<br>Treatment: adjuvant chemotherapy	Fatigue<br>Pain<br>Treatment-related toxicities	NR	Jadad 5-point scale/MINORS: high quality
Mo et al (2021)[48]<br><br>Systematic review	Assess the safety and efficacy of pembrolizumab plus lenvatinib versus their respective monotherapies in solid cancers.	N=4<br>Phase I/II trial<br>Publication range: 2015-2020	N=264 endometrial cancer<br>Cancer type: primary diagnosis, recurrent<br>Treatment: adjuvant chemotherapy	Fatigue<br>Treatment-related toxicities	NR	NR
Shisler et al (2018)[6]<br><br>Systematic review	Summarize the existing literature related to patient reported outcomes among endometrial cancer survivors and highlight gaps in the literature that should be addressed in future research.	N=27<br>19 cross sectional<br>8 longitudinal<br>Publication range: 2005-2017	N=4315<br>Cancer type: stage I-III<br>Treatment: surgery only, adjuvant chemotherapy, radiotherapy, mixed	Fatigue<br>Pain<br>Sexual function<br>Sleep<br>Quality of life<br>Anxiety<br>Depression<br>Distress/stress	BDI<br>BFI<br>BPI<br>BSI-18<br>EORTC-QLQ-C30<br>FACIT-F<br>FAS<br>IDAS<br>PSQI<br>QLACS<br>SF-36<br>SIGH-AD	NR

Physical health: LYMPHOEDEMA

Author (year)	Study aim	Characteristics of primary studies[a]	Characteristics of participants	Health-related outcomes reported[#]	Measurement instruments[a]	Quality appraisal as reported by the review
Lindqvist et al (2017)[25]<br><br>Literature review	Evaluate the prevalence of lower-limb lymphoedema, methods for determining occurrence and the time span until onset of lower-limb lymphoedema symptoms following	N=27<br>2 randomised controlled trial<br>1 prospective<br>24 retrospective<br>Publication range: 1992-2016	N=9031<br>Treatment: mixed	Lymphoedema[#]<br>Quality of life	CaSUN<br>Circumferential measurements<br>EORTC QLQ-C30<br>EORTC QLQ-CX24<br>EORTC QLQ-EN24	NR

	endometrial cancer treatment. Additionally, risk factors for lower-limb lymphoedema and its impact on health quality of life were evaluated.				FACT-En<br>GCLQ-K<br>HADS<br>MRI<br>SF-12<br>Ultrasound<br>Validated lymphoedema questionnaire	
Niikura et al (2019)[60]<br><br>Literature review	Examine and summarise the recently expanding body of literature and discuss sentinel lymph node navigation during surgery in patients with cervical cancer and endometrial cancer.	N=NR<br>Publication range: NR	N=NR<br>Cancer type: primary diagnosis<br>Treatment: adjuvant chemotherapy	Lymphoedema[#]	NR	NR
Zorzato (2020)[61]<br><br>Literature review	Summarize the available evidence on oncologic outcomes between sentinel lymph node mapping and lymph node density in endometrial cancer and analyse the differences in operative complications and long-term complications.	N=10<br>10 retrospective<br>Publication range: 2015-2020	N=2302<br>Cancer type: primary diagnosis, stage I-IV<br>Treatment: adjuvant chemotherapy	Lymphoedema[#]	NR	Quality not assessed, only level of evidence according to Oxford Centre for Evidence-Based Medicine (OCEBM) criteria
**Physical health: OBESITY**						
**Author (year)**	**Study aim**	**Characteristics of primary studies[a]**	**Characteristics of participants**	**Health-related outcomes reported[#]**	**Measurement instruments[a]**	**Quality appraisal as reported by the review**

Alanazi et al (2021)[23]<br><br>Systematic review	Describe the sleep patterns among uterine cancer survivors and verify psychological and physical factors affecting their general quality of life.	N=10<br>4 randomised controlled trial<br>2 prospective<br>1 retrospective<br>2 cross-sectional<br>1 non-randomised controlled trial<br>Publication range: 2015-2019	N=1535 endometrial cancer<br>Cancer type: stage I<br>Treatment: surgery only, radiotherapy, mixed	Fatigue<br>Obesity<br>Pain<br>Sexual function<br>Sleep[#]<br>Quality of life[#]<br>Anxiety<br>Depression	Actigraphy wristwatch + sleep log<br>BMI<br>BSI-18<br>EORTC QLQ-C30<br>FACT-En<br>PSQI<br>PSS<br>QLACS<br>QOL-CS<br>SF-36	Melnyk's evidence pyramid
Bouwman et al (2015)[62]<br><br>Institutional study and systematic review	Evaluate the association between BMI, perioperative complications and outcomes in endometrial cancer.	N=13<br>1 ancillary randomised controlled trial analysis<br>3 prospective<br>8 retrospective<br>1 unknown<br>Publication range: 2003-2015	N=8453<br>Cancer type: primary diagnosis, stage I	Obesity[#]	BMI	NR
Gerli et al (2014)[33]<br><br>Case report and literature review	Present a case of a very young patient with endometrioid adenocarcinoma grade II-III and review the literature on fertility-sparing treatment in endometrial cancer.	N=NR<br>Publication range: 1997-2011	N=NR<br>Treatment: fertility-sparing treatment	Obesity<br>Gravidity<br>Parity	BMI<br>Pregnancy rates<br>Live birth rates	NR
Koskas et al (2014)[19]<br><br>Systematic review and meta-analysis	Evaluate the impact of age, gravidity, obesity, fertility, histology, and hormone treatments on reproductive and oncologic outcomes to better predict the success of fertility-sparing treatment	N=24<br>8 prospective<br>15 retrospective<br>1 unknown<br>Publication range: 2010-2020	N= 266 endometrial cancer<br>Median age: 33 years<br>Age range: 19-44 years<br>Cancer type: stage I-II<br>Treatment: fertility-sparing treatment	Obesity<br>Gravidity	Pregnancy rates	NR

	in endometrial adenocarcinoma and atypical hyperplasia.		Menopausal status: pre-menopausal			
Lee et al (2022)[42]<br><br>Systematic review	Examine the impact of obesity on patient reported sexual health outcomes in women with gynaecological cancer.	N=7<br>3 cross-sectional<br>1 prospective cohort<br>1 retrospective cohort<br>2 trials<br>Publication range: 2015-2020	N=1189 endometrial cancer<br>Mean age: 55-66 years<br>Age range: 28-86 years<br>Cancer type: stage I-IV<br>Treatment: surgery only, adjuvant chemotherapy, radiotherapy, mixed	Obesity[#]<br>Pelvic floor function<br>Sexual function<br>Quality of life	BMI<br>EORTC-QLC-CX24<br>EORTC-QLC-EN24<br>EORTC-QLC-OV28<br>FSFI<br>GUPI<br>PISQ-12<br>QLACS	NOS: high risk of bias, low to moderate quality
Piatek et al (2021)[30]<br><br>Case series and systematic review	Evaluate the treatment results and obstetric outcomes in young women with endometrial cancer/atypical hyperplasia, who underwent fertility-sparing treatment.	N=25<br>4 prospective<br>21 retrospective<br>Publication range: 2001-2021	N=601 endometrial cancer<br>Mean age: 33 years<br>Age range: 18-60 years<br>Cancer type: primary diagnosis, stage I-II<br>Treatment: fertility-sparing treatment, hormone therapy, mixed	Obesity<br>Gravidity<br>Parity	BMI<br>Live birth rates<br>Pregnancy rates	JBI Critical Appraisal Checklist: NR
Russa et al (2021)[21]<br><br>Literature review	Evaluate the outcomes and role of ultra-minimally invasive surgical approaches for treatment of women with endometrial cancer.	N=45<br>17 Prospective<br>28 Retrospective<br>Publication range: 2003-2020	N=1040<br>Age: 20-80 years<br>Cancer type: stage I-II<br>Treatment: Surgery only, hormone therapy, fertility-sparing treatment<br>Menopausal status: Pre-menopausal (n=219) and post-menopausal (n=10)	Obesity<br>Gravidity<br>Parity	Live birth rates<br>Pregnancy rates	NR
**Physical health: PAIN**						

Author (year)	Study aim	Characteristics of primary studies[a]	Characteristics of participants	Health-related outcomes reported[#]	Measurement instruments[a]	Quality appraisal as reported by the review
Alanazi et al (2021)[23]<br><br>Systematic review	Describe the sleep patterns among uterine cancer survivors and verify psychological and physical factors affecting their general quality of life.	N=10<br>4 randomised controlled trial<br>2 prospective<br>1 retrospective<br>2 cross-sectional<br>1 non-randomised controlled trial<br>Publication range: 2015-2019	N=1535 endometrial cancer<br>Cancer type: stage I<br>Treatment: surgery only, radiotherapy, mixed	Fatigue<br>Obesity<br>Pain<br>Sexual function<br>Sleep[#]<br>Quality of life[#]<br>Anxiety<br>Depression	Actigraphy wristwatch + sleep log<br>BMI<br>BSI-18<br>EORTC QLQ-C30<br>FACT-En<br>PSQI<br>PSS<br>QLACS<br>QOL-CS<br>SF-36	Melnyk's evidence pyramid
Burke et al (2014)[44]<br><br>Literature review and practice recommendations	Review the risks and benefits of current treatment options and optimise treatment for women with endometrial cancer through creating evidence-based practice recommendations for diagnosis and treatment.	N=NR	N=NR<br>Treatment: adjuvant chemotherapy, radiotherapy, fertility-sparing treatment	Abdominal discomfort<br>Pain<br>Quality of life[#]	NR	NR
Chen et al (2021)[50]<br><br>Systematic review and meta-analysis	Evaluate the efficacy and safety of bevacizumab-combined chemotherapy in advanced/recurrent endometrial cancer.	N=7<br>2 randomised controlled trial<br>5 single-arm phase II trial<br>Publication range: 2007-2019	N=622<br>Average/median age: 62 years<br>Age range: 57-63 years<br>Cancer type: primary diagnosis, recurrent, stage III-IV<br>Treatment: adjuvant chemotherapy	Fatigue<br>Pain<br>Treatment-related toxicities	NR	Jadad 5-point scale/MINORS: high quality

Hamilton et al (2021)[47]<br><br>Literature review and practice recommendations	Present a comprehensive review of the progress of endometrial cancer treatment.	NR	NR	Pain<br>Sexual health<br>Quality of life[#]	NR	NR
Park et al (2016)[63]<br>Systematic review and meta-analysis	Evaluate the surgical safety and clinic effectiveness of robot-assisted laparoscopic hysterectomy versus conventional laparoscopy and laparotomy for endometrial cancer.	N=37<br>1 non-randomised controlled trial<br>36 observational cohort<br>Publication range: NR	N=3511<br>Mean age: 50-78 years	Pain[#]	Numerical rating scale (0-10)<br>Perioperative clinical data	ROBANS version 2.0: high/unclear risk of bias
Shisler et al (2018)[6]<br><br>Systematic review	Summarize the existing literature related to patient reported outcomes among endometrial cancer survivors and highlight gaps in the literature that should be addressed in future research.	N=27<br>19 cross sectional<br>8 longitudinal<br>Publication range: 2005-2017	N=4315<br>Cancer type: stage I-III<br>Treatment: surgery only, adjuvant chemotherapy, radiotherapy, mixed	Fatigue<br>Pain<br>Sexual function<br>Sleep<br>Quality of life<br>Anxiety<br>Depression<br>Distress/stress	BDI<br>BFI<br>BPI<br>BSI-18<br>EORTC-QLQ-C30<br>FACIT-F<br>FAS<br>IDAS<br>PSQI<br>QLACS<br>SF-36<br>SIGH-AD	NR

Physical health: PELVIC FLOOR FUNCTION

Author (year)	Study aim	Characteristics of primary studies[a]	Characteristics of participants	Health-related outcomes reported[#]	Measurement instruments[a]	Quality appraisal as reported by the review

Brennen et al (2020)[24]<br><br>Systematic review	Identify, evaluate and synthesize the evidence from the studies investigating the effect of non-surgical, non-pharmacological, pelvic floor muscle interventions on any type of pelvic floor dysfunction or health related quality of life in patients following any type of gynaecological cancer treatment.	N=4<br>3 randomised controlled trials<br>1 retrospective<br>Publication range: 1999-2014	N=134 endometrial cancer<br>Mean age: 60 years<br>Age range: 28-84 years<br>Cancer type: stage I-III<br>Treatment: surgery only, adjuvant chemotherapy, radiotherapy, mixed	Abdominal discomfort<br>Pelvic floor function[#]<br>Sexual function<br>Urinary function<br>Quality of life[#]	APFQ<br>CTCAE v.4<br>EORTC QLQ-C30<br>EORTC QLQ-CX24<br>IIQ-SF<br>ISI<br>Motor evoked potential of sacral nerve<br>PFM strength on digital palpation<br>PGI-I<br>QUID<br>SHF<br>SKQ<br>UDI-SF	PEDro Scale (randomised controlled trials): mean score 5/10<br><br>Cochrane risk of bias domains (randomised controlled trials): unclear risk of bias<br><br>NOS (non-randomised studies): mean score 6/9
Lee et al (2022)[42]<br><br>Systematic review	Examine the impact of obesity on patient reported sexual health outcomes in women with gynaecological cancer.	N=7<br>3 cross-sectional<br>1 prospective cohort<br>1 retrospective cohort<br>2 trials<br>Publication range: 2015-2020	N=1189 endometrial cancer<br>Mean age: 55-66 years<br>Age range: 28-86 years<br>Cancer type: stage I-IV<br>Treatment: surgery only, adjuvant chemotherapy, radiotherapy, mixed	Obesity[#]<br>Pelvic floor function<br>Sexual function<br>Quality of life	BMI<br>EORTC-QLC-CX24<br>EORTC-QLC-EN24<br>EORTC-QLC-OV28<br>FSFI<br>GUPI<br>PISQ-12<br>QLACS	NOS: high risk of bias, low to moderate quality
Ramaseshan et al (2018)[64]<br><br>Systematic review	Investigate the prevalence of pelvic floor dysfunctions in cancer survivors after surgical and non-surgical treatments for cervical, uterine, ovarian, and vulvar cancer; and compare rates of pelvic floor dysfunctions among gynaecological cancer survivors and the general population.	N=10<br>3 randomised controlled trials<br>1 prospective<br>6 cross-sectional<br>Publication range: 1990-2013	N=2977 endometrial cancer<br>Median age: 64 years<br>Age range: 34-94 years<br>Cancer type: stage I-IV<br>Treatment: surgery only, adjuvant chemotherapy, radiotherapy, mixed	Pelvic floor function[#]<br>Sexual function	EORTC<br>ICIQ-FLUTS<br>IIQ-7<br>IOC<br>PFDI<br>RSC<br>SSFS<br>SSI<br>SSPQ<br>UDI	Three-category system modified from the AHRQ: Poor quality

Physical health: SARCOPENIA						
**Author (year)**	**Study aim**	**Characteristics of primary studies[a]**	**Characteristics of participants**	**Health-related outcomes reported[#]**	**Measurement instruments[a]**	**Quality appraisal as reported by the review**
Allanson et al (2020)[65]<br><br>Systematic review and meta-analysis	Interrogate the concept of sarcopenia as a prognostic tool for oncological outcomes and for its association with treatment-related complications in gynaecological cancer.	N=4<br>4 retrospective<br>Publication range: 2015-2019	N=804 endometrial cancer<br>Mean age: 64-65 years	Sarcopenia	Average muscle radiation attenuation<br>Muscle mass measurement<br>Skeletal muscle index	Modified NOS: unclear risk of bias
**Physical health: SEXUAL FUNCTION**						
**Author (year)**	**Study aim**	**Characteristics of primary studies[a]**	**Characteristics of participants**	**Health-related outcomes reported[#]**	**Measurement instruments[a]**	**Quality appraisal as reported by the review**
Alanazi et al (2021)[23]<br><br>Systematic review	Describe the sleep patterns among uterine cancer survivors and verify psychological and physical factors affecting their general quality of life.	N=10<br>4 randomised controlled trial<br>2 prospective<br>1 retrospective<br>2 cross-sectional<br>1 non-randomised controlled trial<br>Publication range: 2015-2019	N=1535 endometrial cancer<br>Cancer type: stage I<br>Treatment: surgery only, radiotherapy, mixed	Fatigue<br>Obesity<br>Pain<br>Sexual function<br>Sleep[#]<br>Quality of life[#]<br>Anxiety<br>Depression	Actigraphy wristwatch + sleep log<br>BMI<br>BSI-18<br>EORTC QLQ-C30<br>FACT-En<br>PSQI<br>PSS<br>QLACS<br>QOL-CS<br>SF-36	Melnyk's evidence pyramid
Brennen et al (2020)[24]<br><br>Systematic review	Identify, evaluate and synthesize the evidence from the studies investigating the effect of non-surgical, non-pharmacological, pelvic floor muscle interventions	N=4<br>3 randomised controlled trials<br>1 retrospective<br>Publication range: 1999-2014	N=134 endometrial cancer<br>Mean age: 60 years<br>Age range: 28-84 years<br>Cancer type: stage I-III<br>Treatment: surgery only, adjuvant chemotherapy, radiotherapy, mixed	Abdominal discomfort<br>Pelvic floor function[#]<br>Sexual function<br>Urinary function<br>Quality of life[#]	APFQ<br>CTCAE v.4<br>EORTC QLQ-C30<br>EORTC QLQ-CX24<br>IIQ-SF<br>ISI	PEDro Scale (randomised controlled trials): mean score 5/10<br><br>Cochrane risk of bias domains

	on any type of pelvic floor dysfunction or health related quality of life in patients following any type of gynaecological cancer treatment.				Motor evoked potential of sacral nerve<br>PFM strength on digital palpation<br>PGI-I<br>QUID<br>SHF<br>SKQ<br>UDI-SF	(randomised controlled trials): unclear risk of bias<br><br>NOS (non-randomised studies): mean score 6/9
Hamilton et al (2021)[47]<br><br>Literature review and practice recommendations	Present a comprehensive review of the progress of endometrial cancer treatment.	NR	NR	Pain<br>Sexual health<br>Quality of life[#]	NR	NR
Lee et al (2022)[42]<br><br>Systematic review	Examine the impact of obesity on patient reported sexual health outcomes in women with gynaecological cancer.	N=7<br>3 cross-sectional<br>1 prospective cohort<br>1 retrospective cohort<br>2 trials<br>Publication range: 2015 to 2020	N=1189 endometrial cancer<br>Mean age: 55-66 years<br>Age range: 28-86 years<br>Cancer type: stage I-IV<br>Treatment: surgery only, adjuvant chemotherapy, radiotherapy, mixed	Obesity[#]<br>Pelvic floor function<br>Sexual function<br>Quality of life	BMI<br>EORTC-QLC-CX24<br>EORTC-QLC-EN24<br>EORTC-QLC-OV28<br>FSFI<br>GUPI<br>PISQ-12<br>QLACS	NOS: high risk of bias, low to moderate quality
Mirabeau-Beale et al (2014)[22]<br><br>Literature review	Summarise the literature on quality of life for patients treated with definitive radiation for gynaecological cancer, with a specific focus on patient reported outcomes.	N=8<br>3 randomised controlled trial<br>1 prospective<br>1 retrospective<br>Publication range: NR	N=NR<br>Treatment type: surgery only, adjuvant chemotherapy, radiotherapy, mixed	Abdominal discomfort<br>Sexual function<br>Urinary function<br>Quality of life[#]<br>Treatment-related toxicities	ACOG Sexual Dysfunction Checklist<br>CALGB Sexual Functioning<br>CTCAE<br>EORTC<br>EORTC-CX24<br>EORTC- EN24<br>EORTC QLQ-C30<br>EQ-5D	NR

					FACT<br>FACT-G<br>FSFI<br>GSCQ<br>HADS<br>LENT-SOMA<br>PROMIS<br>QOL-CS<br>RTOG/EORTC late scoring scheme<br>SAQ<br>SF-36<br>WHOQOL-BREF	
Ramaseshan et al (2018)[64]<br><br>Systematic review	Investigate the prevalence of pelvic floor dysfunctions in cancer survivors after surgical and non-surgical treatments for cervical, uterine, ovarian, and vulvar cancer; and compare rates of pelvic floor dysfunctions among gynaecological cancer survivors and the general population.	N=10<br>3 randomised controlled trials<br>1 prospective<br>6 cross-sectional<br>Publication range: 1990-2013	N=2977 endometrial cancer<br>Median age: 64 years<br>Age range: 34-94 years<br>Cancer type: stage I-IV<br>Treatment: surgery only, adjuvant chemotherapy, radiotherapy, mixed	Pelvic floor function[#]<br>Sexual function	EORTC<br>ICIQ-FLUTS<br>IIQ-7<br>IOC<br>PFDI<br>RSC<br>SSFS<br>SSI<br>SSPQ<br>UDI	Three-category system modified from the AHRQ: Poor quality
Shisler et al (2018)[6]<br><br>Systematic review	Summarize the existing literature related to patient reported outcomes among endometrial cancer survivors and highlight gaps in the literature that should be addressed in future research.	N=27<br>19 cross sectional<br>8 longitudinal<br>Publication range: 2005-2017	N=4315<br>Cancer type: stage I-III<br>Treatment: surgery only, adjuvant chemotherapy, radiotherapy, mixed	Fatigue<br>Pain<br>Sexual function<br>Sleep<br>Quality of life<br>Anxiety<br>Depression<br>Distress/stress	BDI<br>BFI<br>BPI<br>BSI-18<br>EORTC-QLQ-C30<br>FACIT-F<br>FAS<br>IDAS<br>PSQI<br>QLACS<br>SF-36<br>SIGH-AD	NR

White et al (2016)[43]<br><br>Systematic review	Critically appraise the measurement properties and clinical utility of instruments validated for the measurement of female sexual dysfunction among cervical cancer or endometrial cancer patients.	N=3<br>NR<br>Publication range: 2011-2012	N=NR<br>Age range: 18-87 years	Sexual function[#]<br>Quality of Life	EORTC QLQ CX-24<br>EORTC QLQ-EN24<br>FSFI<br>GLQ<br>SABIS-G<br>SVQ	COSMIN Checklist: Good (n=1), Fair (n=1), excellent (n=2) and poor (n=2)
**Physical health: SLEEP**						
**Author (year)**	**Study aim**	**Characteristics of primary studies[a]**	**Characteristics of participants**	**Health-related outcomes reported[#]**	**Measurement instruments[a]**	**Quality appraisal as reported by the review**
Alanazi et al (2021)[23]<br><br>Systematic review	Describe the sleep patterns among uterine cancer survivors and verify psychological and physical factors affecting their general quality of life.	N=10<br>4 randomised controlled trial<br>2 prospective<br>1 retrospective<br>2 cross-sectional<br>1 non-randomised controlled trial<br>Publication range: 2015-2019	N=1535 endometrial cancer<br>Cancer type: stage I<br>Treatment: surgery only, radiotherapy, mixed	Fatigue<br>Obesity<br>Pain<br>Sexual function<br>Sleep[#]<br>Quality of life[#]<br>Anxiety<br>Depression	Actigraphy wristwatch + sleep log<br>BMI<br>BSI-18<br>EORTC QLQ-C30<br>FACT-En<br>PSQI<br>PSS<br>QLACS<br>QOL-CS<br>SF-36	Melnyk's evidence pyramid
Shisler et al (2018)[6]<br><br>Systematic review	Summarize the existing literature related to patient reported outcomes among endometrial cancer survivors and highlight gaps in the literature that should be addressed in future research.	N=27<br>19 cross sectional<br>8 longitudinal<br>Publication range: 2005-2017	N=4315<br>Cancer type: stage I-III<br>Treatment: surgery only, adjuvant chemotherapy, radiotherapy, mixed	Fatigue<br>Pain<br>Sexual function<br>Sleep<br>Quality of life<br>Anxiety<br>Depression<br>Distress/stress	BDI<br>BFI<br>BPI<br>BSI-18<br>EORTC-QLQ-C30<br>FACIT-F<br>FAS<br>IDAS<br>PSQI<br>QLACS	NR

					SF-36<br>SIGH-AD	

Physical health: URINARY FUNCTION

Author (year)	Study aim	Characteristics of primary studies[a]	Characteristics of participants	Health-related outcomes reported[#]	Measurement instruments[a]	Quality appraisal as reported by the review
Brennen et al (2020)[24]<br><br>Systematic review	Identify, evaluate and synthesize the evidence from the studies investigating the effect of non-surgical, non-pharmacological, pelvic floor muscle interventions on any type of pelvic floor dysfunction or health related quality of life in patients following any type of gynaecological cancer treatment.	N=4<br>3 randomised controlled trials<br>1 retrospective<br>Publication range: 1999-2014	N=134 endometrial cancer<br>Mean age: 60 years<br>Age range: 28-84 years<br>Cancer type: stage I-III<br>Treatment: surgery only, adjuvant chemotherapy, radiotherapy, mixed	Abdominal discomfort<br>Pelvic floor function[#]<br>Sexual function<br>Urinary function<br>Quality of life[#]	APFQ<br>CTCAE v.4<br>EORTC QLQ-C30<br>EORTC QLQ-CX24<br>IIQ-SF<br>ISI<br>Motor evoked potential of sacral nerve<br>PFM strength on digital palpation<br>PGI-I<br>QUID<br>SHF<br>SKQ<br>UDI-SF	PEDro Scale (randomised controlled trials): mean score 5/10<br><br>Cochrane risk of bias domains (randomised controlled trials): unclear risk of bias<br><br>NOS (non-randomised studies): mean score 6/9
Mirabeau-Beale et al (2014)[22]<br><br>Literature review	Summarise the literature on quality of life for patients treated with definitive radiation for gynaecological cancer, with a specific focus on patient reported outcomes.	N=8<br>3 randomised controlled trial<br>1 prospective<br>1 retrospective<br>Publication range: NR	N=NR<br>Treatment type: surgery only, adjuvant chemotherapy, radiotherapy, mixed	Abdominal discomfort<br>Sexual function<br>Urinary function<br>Quality of life[#]<br>Treatment-related toxicities	ACOG Sexual Dysfunction Checklist<br>CALGB Sexual Functioning<br>CTCAE<br>EORTC<br>EORTC-CX24	NR

					EORTC- EN24<br>EORTC QLQ-C30<br>EQ-5D<br>FACT<br>FACT-G<br>FSFI<br>GSCQ<br>HADS<br>LENT-SOMA<br>PROMIS<br>QOL-CS<br>RTOG/EORTC late scoring scheme<br>SAQ<br>SF-36<br>WHOQOL-BREF	
**Fertility Outcomes: GRAVIDITY**						
**Author (year)**	**Study aim**	**Characteristics of primary studies[a]**	**Characteristics of participants**	**Health-related outcomes reported[#]**	**Measurement instruments[a]**	**Quality appraisal as reported by the review**
Arendas et al (2015)[35]<br><br>Case series and literature review	Describe two cases of stage IA endometrial cancer managed conservatively by hysteroscopic surgery and medical therapy for fertility-sparing purposes, and review the literature on the use of hysteroscopic resection in conservative management of endometrial cancer to preserve fertility.	N=3<br>2 prospective<br>1 case series<br>Publication range: 2010-2015	N=22<br>Cancer type: primary diagnosis, stage I<br>Treatment: surgery only, hormone therapy, fertility-sparing treatment, mixed	Gravidity	Pregnancy rates	NA
Coakley et al (2019)[39]	Review the recent research regarding fertility-sparing treatment of endometrial	N=5<br>1 Meta-analysis<br>3 Retrospective	N=696 endometrial cancer<br>Treatment: fertility-sparing treatment, mixed	Gravidity<br>Parity	Pregnancy rates<br>Live birth rates	NA

Literature review	cancer, cervical, and ovarian cancer in the context of current standards of care and evaluate the pregnancy outcomes and uptake of these treatments.	1 pilot study<br>Publication range: 2018-2019				
Garzon et al (2021)[31]<br><br>Literature review	Provide an overview of the available evidence on different fertility-sparing treatment options in endometrial cancer, specifically their effect on oncologic and reproductive outcomes.	N=78<br>69 clinical studies<br>9 systematic review and meta-analysis<br>Publication range: NR	N=NR<br>Treatment: fertility-sparing treatment	Gravidity<br>Parity	Live birth rates<br>Pregnancy rates	NR
Gerli et al (2014)[33]<br><br>Case report and literature review	Present a case of a very young patient with endometrioid adenocarcinoma grade II-III and review the literature on fertility-sparing treatment in endometrial cancer.	N=NR<br>Publication range: 1997-2011	N=NR<br>Treatment: fertility-sparing treatment	Obesity<br>Gravidity<br>Parity	BMI<br>Pregnancy rates<br>Live birth rates	NR
Gerstl et al (2019)[18]<br><br>Systematic review	Report on the reproductive outcomes following gynaecological cancer diagnosis in pre-menopausal women and provide information to assist cancer and reproductive health professionals in discussing fertility preservation options with patients following treatment.	N=12<br>4 prospective<br>5 retrospective<br>3 cohort<br>Publication range: 1997-2016	N=376 endometrial cancer<br>Menopausal status: pre-menopausal	Gravidity<br>Parity	Pregancy rates<br>Live birth rates	NR

Giampaolino et al (2022)[27]<br><br>Systematic review	Provide an overview of conservative management of endometroid endometrial cancer grade 2 stage 1A and to analyse the oncological and reproductive outcomes before and after conservative hormone therapy.	N=12<br>1 prospective<br>7 retrospective<br>4 case report<br>Publication range: 2010-2020	N=84<br>Age range: 13-85 years<br>Cancer type: endometrial cancer grade 2 stage 1A<br>Treatment: hormone therapy, fertility-sparing treatment	Gravidity<br>Parity	Pregnancy rates<br>Live birth rates	JBI Critical Appraisal Tool: low risk of bias
Herrera Cappelletti et al (2022)[28]<br><br>Systematic review and meta-analysis	Evaluate the reproductive outcomes of conservative management of endometrial cancer.	N=46<br>22 cohort<br>24 case series<br>Publication range: 2001-2021	N=861<br>Median age: 25-39 years<br>Treatment: fertility-sparing treatment	Gravidity<br>Parity	Live birth rates	Single-Arm Study Quality Assessment: mean score 6.6/10
Koskas et al (2014)[19]<br><br>Systematic review and meta-analysis	Evaluate the impact of age, gravidity, obesity, fertility, histology, and hormone treatments on reproductive and oncologic outcomes to better predict the success of fertility-sparing treatment in endometrial adenocarcinoma and atypical hyperplasia.	N=24<br>8 prospective<br>15 retrospective<br>1 unknown<br>Publication range: 2010-2020	N= 266 endometrial cancer<br>Median age: 33 years<br>Age range: 19-44 years<br>Cancer type: stage I-II<br>Treatment: fertility-sparing treatment<br>Menopausal status: pre-menopausal	Obesity<br>Gravidity	Pregnancy rates	NR
Laurelli et al (2015)[34]<br><br>Institutional study and literature review	Present the disease outcome of low-grade endometrial stromal sarcoma in six young women conservatively treated by combined hysteroscopic resection and hormone therapy; and review the limited literature available.	N=13<br>Majority case reports<br>Publication range: 2005-2015	N=30 endometrial cancer<br>Age range: 16-40 years<br>Cancer type: primary diagnosis, stage I<br>Treatment: surgery only, adjuvant chemotherapy, hormone therapy, fertility-sparing treatment, mixed	Gravidity<br>Parity	Live birth rates<br>Pregnancy rates	NA

Murakami (2023)[26]<br><br>Systematic review	Examine the effectiveness of progestin re-treatment for recurrent endometrial intraepithelial neoplasia, atypical hyperplasia and endometrial cancer following initial fertility-sparing treatment.	N=32<br>11 prospective<br>21 retrospective<br>Publication range: 2001-2022	N=293<br>Median age: 33 years<br>Age range: 29-37 years<br>Cancer type: recurrent<br>Treatment: adjuvant chemotherapy, surgery only, fertility-sparing treatment	Gravidity<br>Parity	BMI<br>Pregnancy rates<br>Live birth rates	ROBINS-I: serious risk of bias n=11; moderate risk of bias n=21
Piatek et al (2021)[30]<br><br>Case series and systematic review	Evaluate the treatment results and obstetric outcomes in young women with endometrial cancer/atypical hyperplasia, who underwent fertility-sparing treatment.	N=25<br>4 prospective<br>21 retrospective<br>Publication range: 2001-2021	N=601 endometrial cancer<br>Mean age: 33 years<br>Age range: 18-60 years<br>Cancer type: primary diagnosis, stage I-II<br>Treatment: fertility-sparing treatment, hormone therapy, mixed	Obesity<br>Gravidity<br>Parity	BMI<br>Live birth rates<br>Pregnancy rates	JBI Critical Appraisal Checklist: NR
Prodromidou et al (2021)[20]<br><br>Systematic review and meta-analysis	Evaluate the effect of metformin and progesterone monotherapy on preserving fertility in endometrial cancer patients and examine the potential preventive role of metformin in breast cancer survivors and obese patients.	N=2<br>2 randomised controlled trial<br>Publication range: 2020	N=205 endometrial cancer<br>Mean age: 44 years<br>Cancer type: primary diagnosis, recurrent<br>Treatment: adjuvant chemotherapy, fertility-sparing treatment<br>Menopausal status: post-menopausal (n=32)	Abdominal pain<br>Gravidity<br>Parity<br>Treatment-related toxicities	Pregnancy rates<br>Live birth rates	Cochrane Risk of Bias tool: low risk of bias
Russa et al (2021)[21]<br><br>Literature review	Evaluate the outcomes and role of ultra-minimally invasive surgical approaches for treatment of women with endometrial cancer.	N=45<br>17 Prospective<br>28 Retrospective<br>Publication range: 2003-2020	N=1040<br>Age: 20-80 years<br>Cancer type: stage I-II<br>Treatment: Surgery only, hormone therapy, fertility-sparing treatment<br>Menopausal status: Pre-menopausal (n=219) and post-menopausal (n=10)	Obesity<br>Gravidity<br>Parity	Live birth rates<br>Pregnancy rates	NR

Tanos et al (2022)[37]<br><br>Systematic review	Identify and summarise the currently established biomolecular and genetic prognostic factors that can facilitate decision making for fertility-sparing treatment in early-stage endometrial cancer.	N=34<br>Study type NR<br>Publication range: 1998-2021	N=9165<br>Cancer type: primary diagnosis, recurrent diagnosis, stage I-II<br>Treatment: surgery only, hormone therapy, fertility-sparing treatment, mixed	Gravidity<br>Parity	Live birth rates<br>Pregnancy rates	CASP: results NR
Trojano et al (2019)[36]<br><br>Literature review	Determine the strategies and risks associated with conservative treatment of early-stage endometrial cancer and obstetric outcomes following treatment.	N=NR<br>Case reports<br>Case series<br>Original articles<br>Review articles<br>Meta-analyses<br>Publication range: NR	N=NR<br>Cancer type: primary diagnosis, stage I<br>Treatment: surgery only, adjuvant chemotherapy, radiotherapy, fertility-sparing treatment, mixed	Gravidity	NA	NA
Wei et al (2017)[32]<br><br>Systematic review and meta-analysis	Compare fertility-sparing treatments by evaluating the oncologic and reproductive outcomes in patients with endometrial cancer or atypical complex hyperplasia.	N=12<br>17 retrospective<br>11 prospective<br>Publication range: 1997-2016	N=320 endometrial cancer<br>Age range: 28-58 years<br>Cancer type: primary diagnosis<br>Treatment: hormone therapy, fertility-sparing treatment	Gravidity<br>Parity	Live birth rates<br>Pregnancy rates	Modified 18-item Delphi Checklist: good quality
Zhao et al (2021)[40]<br><br>Systematic review and meta-analysis	Investigate the efficacies of different methods on fertility preservation in patients with early-stage endometrial cancer.	N=23<br>Cohort<br>Case-series<br>Publication range: 2003-2019	N=446<br>Age range: 18-40 years<br>Cancer type: stage I<br>Treatment: fertility-sparing treatment, mixed	Gravidity<br>Parity	Live birth rates<br>Pregnancy rates	MINORS scale items 1-8: moderate quality
Zisi et al (2022)[29]<br><br>Literature review	Present all recent data on uterine sparing strategies in patients with endometrial cancer or atypical hyperplasia, to help clinicians make the correct	N=NR<br>Publication range: 2009-2021	NR	Gravidity<br>Parity	BMI<br>Live birth rates<br>Pregnancy rates	NR

	patient selection, treatment and follow-up strategy, according to personalised patient evaluation.					
**Fertility Outcomes: PARITY**						
**Author (year)**	**Study aim**	**Characteristics of primary studies[a]**	**Characteristics of participants**	**Health-related outcomes reported[#]**	**Measurement instruments[a]**	**Quality appraisal as reported by the review**
Coakley et al (2019)[39]<br><br>Literature review	Review the recent research regarding fertility-sparing treatment of endometrial cancer, cervical, and ovarian cancer in the context of current standards of care and evaluate the pregnancy outcomes and uptake of these treatments.	N=5<br>1 Meta-analysis<br>3 Retrospective<br>1 pilot study<br>Publication range: 2018-2019	N=696 endometrial cancer<br>Treatment: fertility-sparing treatment, mixed	Gravidity<br>Parity	Pregnancy rates<br>Live birth rates	NA
Garzon et al (2021)[31]<br><br>Literature review	Provide an overview of the available evidence on different fertility-sparing treatment options in endometrial cancer, specifically their effect on oncologic and reproductive outcomes.	N=78<br>69 clinical studies<br>9 systematic review and meta-analysis<br>Publication range: NR	N=NR<br>Treatment: fertility-sparing treatment	Gravidity<br>Parity	Live birth rates<br>Pregnancy rates	NR
Gerli et al (2014)[33]<br><br>Case report and literature review	Present a case of a very young patient with endometrioid adenocarcinoma grade II-III and review the literature on fertility-sparing treatment in endometrial cancer.	N=NR<br>Publication range: 1997-2011	N=NR<br>Treatment: fertility-sparing treatment	Obesity<br>Gravidity<br>Parity	BMI<br>Pregnancy rates<br>Live birth rates	NR
Gerstl et al (2019)[18]	Report on the reproductive outcomes following	N=12<br>4 prospective	N=376 endometrial cancer	Gravidity<br>Parity	Pregancy rates<br>Live birth rates	NR

Systematic review	gynaecological cancer diagnosis in pre-menopausal women and provide information to assist cancer and reproductive health professionals in discussing fertility preservation options with patients following treatment.	5 retrospective<br>3 cohort<br>Publication range: 1997-2016	Menopausal status: pre-menopausal			
Giampaolino et al (2022)[27]<br><br>Systematic review	Provide an overview of conservative management of endometroid endometrial cancer grade 2 stage 1A and to analyse the oncological and reproductive outcomes before and after conservative hormone therapy.	N=12<br>1 prospective<br>7 retrospective<br>4 case report<br>Publication range: 2010-2020	N=84<br>Age range: 13-85 years<br>Cancer type: endometrial cancer grade 2 stage 1A<br>Treatment: hormone therapy, fertility-sparing treatment	Gravidity<br>Parity	Pregnancy rates<br>Live birth rates	JBI Critical Appraisal Tool: low risk of bias
Herrera Cappelletti et al (2022)[28]<br><br>Systematic review and meta-analysis	Evaluate the reproductive outcomes of conservative management of endometrial cancer.	N=46<br>22 cohort<br>24 case series<br>Publication range: 2001-2021	N=861<br>Median age: 25-39 years<br>Treatment: fertility-sparing treatment	Gravidity<br>Parity	Live birth rate	Single-Arm Study Quality Assessment: mean score 6.6/10
Laurelli et al (2015)[34]<br><br>Institutional study and literature review	Present the disease outcome of low-grade endometrial stromal sarcoma in six young women conservatively treated by combined hysteroscopic resection and hormone therapy; and review the limited literature available.	N=13<br>Majority case reports<br>Publication range: 2005-2015	N=30 endometrial cancer<br>Age range: 16-40 years<br>Cancer type: primary diagnosis, stage I<br>Treatment: surgery only, adjuvant chemotherapy, hormone therapy, fertility-sparing treatment, mixed	Gravidity<br>Parity	Live birth rates<br>Pregnancy rates	NA

McKenzie et al (2018)[38]<br><br>Literature review	Provide an update on fertility-preserving in gynaecological cancer as well as those with genetic predisposition for gynaecological cancer.	N=13<br>Publication range: 1986-2013	N=240 endometrial cancer<br>Treatment: adjuvant chemotherapy, radiotherapy, fertility-sparing treatment	Parity	Live birth rates	NA
Murakami (2023)[26]<br><br>Systematic review	Examine the effectiveness of progestin re-treatment for recurrent endometrial intraepithelial neoplasia, atypical hyperplasia and endometrial cancer following initial fertility-sparing treatment.	N=32<br>11 prospective<br>21 retrospective<br>Publication range: 2001-2022	N=293<br>Median age: 33 years<br>Age range: 29-37 years<br>Cancer type: recurrent<br>Treatment: adjuvant chemotherapy, surgery only, fertility-sparing treatment	Gravidity<br>Parity	Pregnancy rates<br>Live birth rates	ROBINS-I: serious risk of bias n=11; moderate risk of bias n=21
Piatek et al (2021)[30]<br><br>Case series and systematic review	Evaluate the treatment results and obstetric outcomes in young women with endometrial cancer/atypical hyperplasia, who underwent fertility-sparing treatment.	N=25<br>4 prospective<br>21 retrospective<br>Publication range: 2001-2021	N=601 endometrial cancer<br>Mean age: 33 years<br>Age range: 18-60 years<br>Cancer type: primary diagnosis, stage I-II<br>Treatment: fertility-sparing treatment, hormone therapy, mixed	Obesity<br>Gravidity<br>Parity	BMI<br>Live birth rates<br>Pregnancy rates	JBI Critical Appraisal Checklist: NR
Prodromidou et al (2021)[20]<br><br>Systematic review and meta-analysis	Evaluate the effect of metformin and progesterone monotherapy on preserving fertility in endometrial cancer patients and examine the potential preventive role of metformin in breast cancer survivors and obese patients.	N=2<br>2 randomised controlled trial<br>Publication range: 2020	N=205 endometrial cancer<br>Mean age: 44 years<br>Cancer type: primary diagnosis, recurrent<br>Treatment: adjuvant chemotherapy, fertility-sparing treatment<br>Menopausal status: post-menopausal (n=32)	Abdominal pain<br>Gravidity<br>Parity<br>Treatment-related toxicities	Pregnancy rates<br>Live birth rates	Cochrane Risk of Bias tool: low risk of bias
Russa et al (2021)[21]	Evaluate the outcomes and role of ultra-minimally	N=45<br>17 Prospective	N=1040<br>Age: 20-80 years	Obesity<br>Gravidity	Live birth rates<br>Pregnancy rates	NR

Literature review	invasive surgical approaches for treatment of women with endometrial cancer.	28 Retrospective<br>Publication range: 2003-2020	Cancer type: stage I-II<br>Treatment: Surgery only, hormone therapy, fertility-sparing treatment<br>Menopausal status: Pre-menopausal (n=219) and post-menopausal (n=10)	Parity		
Suri (2015)[41]<br><br>Literature review	Review the current literature on management of endometrial cancer, including imaging techniques for metastatic diagnosis and current treatment options.	N=NR<br>Randomised controlled trial<br>Meta-analysis<br>Cochrane reviews<br>Publication range: NR	N=NR<br>Cancer type: primary, recurrent, stage I-IV<br>Treatment: surgery only, adjuvant chemotherapy, radiotherapy, fertility-sparing treatment, mixed	Parity	Live birth rates	NR
Tanos et al (2022)[37]<br><br>Systematic review	Identify and summarise the currently established biomolecular and genetic prognostic factors that can facilitate decision making for fertility-sparing treatment in early-stage endometrial cancer.	N=34<br>Study type NR<br>Publication range: 1998-2021	N=9165<br>Cancer type: primary diagnosis, recurrent diagnosis, stage I-II<br>Treatment: surgery only, hormone therapy, fertility-sparing treatment, mixed	Gravidity<br>Parity	Live birth rates<br>Pregnancy rates	CASP: results NR
Wei et al (2017)[32]<br><br>Systematic review and meta-analysis	Compare fertility-sparing treatments by evaluating the oncologic and reproductive outcomes in patients with endometrial cancer or atypical complex hyperplasia.	N=12<br>17 retrospective<br>11 prospective<br>Publication range: 1997-2016	N=320<br>Age range: 28-58 years<br>Cancer type: primary diagnosis<br>Treatment: hormone therapy, fertility-sparing treatment	Gravidity<br>Parity	Live birth rates<br>Pregnancy rates	Modified 18-item Delphi Checklist: good quality
Zhao et al (2021)[40]<br><br>Systematic review and meta-analysis	Investigate the efficacies of different methods on fertility preservation in	N=23<br>Cohort<br>Case-series	N=446<br>Age range: 18-40 years<br>Cancer type: stage I	Gravidity<br>Parity	Live birth rates<br>Pregnancy rates	MINORS scale items 1-8: moderate quality

	patients with early-stage endometrial cancer.	Publication range: 2003-2019	Treatment: fertility-sparing treatment, mixed			
Zisi et al (2022)[29]<br><br>Literature review	Present all recent data on uterine sparing strategies in patients with endometrial cancer or atypical hyperplasia, to help clinicians make the correct patient selection, treatment and follow-up strategy, according to personalised patient evaluation.	N=NR<br>Publication range: 2009-2021	NR	Gravidity<br>Parity	BMI<br>Live birth rates<br>Pregnancy rates	NR
**QUALITY OF LIFE**						
**Author (year)**	**Study aim**	**Characteristics of primary studies[a]**	**Characteristics of participants**	**Health-related outcomes reported[#]**	**Measurement instruments[a]**	**Quality appraisal as reported by the review**
Alanazi et al (2021)[23]<br><br>Systematic review	Describe the sleep patterns among uterine cancer survivors and verify psychological and physical factors affecting their general quality of life.	N=10<br>4 randomised controlled trial<br>2 prospective<br>1 retrospective<br>2 cross-sectional<br>1 non-randomised controlled trial<br>Publication range: 2015-2019	N=1535 endometrial cancer<br>Cancer type: stage I<br>Treatment: surgery only, radiotherapy, mixed	Fatigue<br>Obesity<br>Pain<br>Sexual function<br>Sleep[#]<br>Quality of life[#]<br>Anxiety<br>Depression	Actigraphy wristwatch + sleep log<br>BMI<br>BSI-18<br>EORTC QLQ-C30<br>FACT-En<br>PSQI<br>PSS<br>QLACS<br>QOL-CS<br>SF-36	Melnyk's evidence pyramid
Brennen et al (2020)[24]<br><br>Systematic review	Identify, evaluate and synthesize the evidence from the studies investigating the effect of non-surgical, non-pharmacological, pelvic	N=4<br>3 randomised controlled trials<br>1 retrospective<br>Publication range: 1999-2014	N=134 endometrial cancer<br>Mean age: 60 years<br>Age range: 28-84 years<br>Cancer type: stage I-III	Abdominal discomfort<br>Pelvic floor function[#]<br>Sexual function<br>Urinary function<br>Quality of life[#]	APFQ<br>CTCAE v.4<br>EORTC QLQ-C30<br>EORTC QLQ-CX24<br>IIQ-SF<br>ISI	PEDro Scale (randomised controlled trials): mean score 5/10

	floor muscle interventions on any type of pelvic floor dysfunction or health related quality of life in patients following any type of gynaecological cancer treatment.		Treatment: surgery only, adjuvant chemotherapy, radiotherapy, mixed		Motor evoked potential of sacral nerve<br>PFM strength on digital palpation<br>PGI-I<br>QUID<br>SHF<br>SKQ<br>UDI-SF	Cochrane risk of bias domains (randomised controlled trials): unclear risk of bias<br><br>NOS (non-randomised studies): mean score 6/9
Burke et al (2014)[44]<br><br>Literature review and practice recommendations	Review the risks and benefits of current treatment options and optimise treatment for women with endometrial cancer through creating evidence-based practice recommendations for diagnosis and treatment.	N=NR	N=NR<br>Treatment: adjuvant chemotherapy, radiotherapy, fertility-sparing treatment	Abdominal discomfort<br>Pain<br>Quality of life[#]	NR	NR
Hamilton et al (2021)[47]<br><br>Literature review and practice recommendations	Present a comprehensive review of the progress of endometrial cancer treatment.	NR	NR	Pain<br>Sexual health<br>Quality of life[#]	NR	NR
He et al (2013)[45]<br><br>Systematic review	Assess the efficacy and safety of laparoscopy compared with laparotomy for treatment of endometrial cancer.	N=9<br>9 randomised controlled trial<br>Publication range: 2005-2011	N=3616<br>Treatment: surgery only	Quality of life	EQ-5D<br>FACT-G<br>Kupperman Index<br>QoR-40<br>RAND-36<br>RI10<br>SF-36	Cochrane Handbook for Systematic Reviews of Interventions: moderate quality
Lee et al (2022)[42]<br><br>Systematic review	Examine the impact of obesity on patient reported sexual health outcomes in	N=7<br>3 cross-sectional	N=1189 endometrial cancer<br>Mean age: 55-66 years<br>Age range: 28-86 years	Obesity[#]<br>Pelvic floor function<br>Sexual function	BMI<br>EORTC-QLC-CX24<br>EORTC-QLC-EN24	NOS: high risk of bias, low to moderate quality

	women with gynaecological cancer.	1 prospective cohort<br>1 retrospective cohort<br>2 trials<br>Publication range: 2015-2020	Cancer type: stage I-IV<br>Treatment: surgery only, adjuvant chemotherapy, radiotherapy, mixed	Quality of life	EORTC-QLC-OV28<br>FSFI<br>GUPI<br>PISQ-12<br>QLACS	
Lindqvist et al (2017)[25]<br><br>Literature review	Evaluate the prevalence of lower-limb lymphoedema, methods for determining occurrence and the time span until onset of lower-limb lymphoedema symptoms following endometrial cancer treatment. Additionally, risk factors for lower-limb lymphoedema and its impact on health quality of life were evaluated.	N=27<br>2 randomised controlled trial<br>1 prospective<br>24 retrospective<br>Publication range: 1992-2016	N=9031<br>Treatment: mixed	Lymphoedema[#]<br>Quality of life	CaSUN<br>Circumferential measurements<br>EORTC QLQ-C30<br>EORTC QLQ-CX24<br>EORTC QLQ-EN24<br>FACT-En<br>GCLQ-K<br>HADS<br>MRI<br>SF-12<br>Ultrasound<br>Validated lymphoedema questionnaire	NR
Mirabeau-Beale et al (2014)[22]<br><br>Literature review	Summarise the literature on quality of life for patients treated with definitive radiation for gynaecological cancer, with a specific focus on patient reported outcomes.	N=8<br>3 randomised controlled trial<br>1 prospective<br>1 retrospective<br>Publication range: NR	N=NR<br>Treatment type: surgery only, adjuvant chemotherapy, radiotherapy, mixed	Abdominal discomfort<br>Sexual function<br>Urinary function<br>Quality of life[#]<br>Treatment-related toxicities	ACOG Sexual Dysfunction Checklist<br>CALGB Sexual Functioning<br>CTCAE<br>EORTC<br>EORTC-CX24<br>EORTC- EN24<br>EORTC QLQ-C30<br>EQ-5D<br>FACT<br>FACT-G<br>FSFI<br>GSCQ	NR

					HADS<br>LENT-SOMA<br>PROMIS<br>QOL-CS<br>RTOG/EORTC late scoring scheme<br>SAQ<br>SF-36<br>WHOQOL-BREF	
Moss et al (2023)[46]<br><br>Systematic review	Provide perspective on patient-reported outcome measures to adopt in patients diagnosed with gynaecological cancer.	N=31<br>Study type NR<br>Publication range: 2001-2020	N=739<br>Mean age: 63 years<br>Age range: 30-88 years<br>Cancer type: primary diagnosis, stage I-IV	Quality of life[#]	EORTC QLQ-C30<br>EORTC QLQ-EN24<br>FACT-GOG-Ntx	COSMIN risk of bias checklist: Good quality
Shisler et al (2018)[6]<br><br>Systematic review	Summarize the existing literature related to patient reported outcomes among endometrial cancer survivors and highlight gaps in the literature that should be addressed in future research.	N=27<br>19 cross sectional<br>8 longitudinal<br>Publication range: 2005-2017	N=4315<br>Cancer type: stage I-III<br>Treatment: surgery only, adjuvant chemotherapy, radiotherapy, mixed	Fatigue<br>Pain<br>Sexual function<br>Sleep<br>Quality of life<br>Anxiety<br>Depression<br>Distress/stress	BDI<br>BFI<br>BPI<br>BSI-18<br>EORTC-QLQ-C30<br>FACIT-F<br>FAS<br>IDAS<br>PSQI<br>QLACS<br>SF-36<br>SIGH-AD	NR
White et al (2016)[43]<br><br>Systematic review	Critically appraise the measurement properties and clinical utility of instruments validated for the measurement of female sexual dysfunction among cervical cancer or endometrial cancer patients.	N=3<br>NR<br>Publication range: 2011-2012	N=NR<br>Age range: 18-87 years	Sexual function[#]<br>Quality of Life	EORTC QLQ CX-24<br>EORTC QLQ-EN24<br>FSFI<br>GLQ<br>SABIS-G<br>SVQ	COSMIN Checklist: Good (n=1), Fair (n=1), excellent (n=2) and poor (n=2)

Other: TREATMENT-RELATED TOXICITIES						
**Author (year)**	**Study aim**	**Characteristics of primary studies[a]**	**Characteristics of participants**	**Health-related outcomes reported[#]**	**Measurement instruments[a]**	**Quality appraisal as reported by the review**
Chen et al (2021)[50]<br><br>Systematic review and meta-analysis	Evaluate the efficacy and safety of bevacizumab-combined chemotherapy in advanced/recurrent endometrial cancer.	N=7<br>2 randomised controlled trial<br>5 single-arm phase II trial<br>Publication range: 2007-2019	N=622<br>Average/median age: 62 years<br>Age range: 57-63 years<br>Cancer type: primary diagnosis, recurrent, stage III-IV<br>Treatment: adjuvant chemotherapy	Fatigue<br>Pain<br>Treatment-related toxicities	NR	Jadad 5-point scale/MINORS: high quality
Charo et al (2019)[51]<br><br>Literature review	Highlight recent advances in endometrial cancer research, focusing on surgical staging, sentinel lymph node mapping, adjuvant treatment, combination therapy, molecular biology and immunotherapy.	N=7<br>Phase III clinical trials<br>Publication range: 2015-2019	N=3923<br>Cancer type: primary diagnosis, recurrent, stage I-IV<br>Treatment: adjuvant chemotherapy, radiotherapy, mixed	Fatigue<br>Treatment-related toxicities	FACIT	NR
Kassem et al (2016)[49]<br><br>Systematic review	Review the current evidence that supports the expansion of using mTOR inhibitors in the treatment of advanced GC.	N=11<br>Phase I/II trial<br>Publication range: 2010-2015	N=571 endometrial cancer<br>Median age: 64 years<br>Age range: 58-73 years<br>Cancer type: primary diagnosis, recurrent, stage III-IV<br>Treatment: adjuvant chemotherapy	Treatment-related toxicities	CTCAE	Not completed: Planned to use Cochrane RoB but not feasible as most studies were non-randomised
Mirabeau-Beale et al (2014)[22]<br><br>Literature review	Summarise the literature on quality of life for patients treated with definitive radiation for gynaecological cancer, with a specific focus on patient reported outcomes.	N=8<br>3 randomised controlled trial<br>1 prospective<br>1 retrospective<br>Publication range: NR	N=NR<br>Treatment type: surgery only, adjuvant chemotherapy, radiotherapy, mixed	Abdominal discomfort<br>Sexual function<br>Urinary function<br>Quality of life[#]<br>Treatment-related toxicities	ACOG Sexual Dysfunction Checklist<br>CALGB Sexual Functioning<br>CTCAE<br>EORTC<br>EORTC-CX24	NR

					EORTC- EN24<br>EORTC QLQ-C30<br>EQ-5D<br>FACT<br>FACT-G<br>FSFI<br>GSCQ<br>HADS<br>LENT-SOMA<br>PROMIS<br>QOL-CS<br>RTOG/EORTC late scoring scheme<br>SAQ<br>SF-36<br>WHOQOL-BREF	
Mo et al (2021)[48]<br><br>Systematic review	Assess the safety and efficacy of pembrolizumab plus lenvatinib versus their respective monotherapies in solid cancers.	N=4<br>Phase I/II trial<br>Publication range: 2015-2020	N=264 endometrial cancer<br>Cancer type: primary diagnosis, recurrent<br>Treatment: adjuvant chemotherapy	Fatigue<br>Treatment-related toxicities	NR	NR
Prodromidou et al (2021)[20]<br><br>Systematic review and meta-analysis	Evaluate the effect of metformin and progesterone monotherapy on preserving fertility in endometrial cancer patients and examine the potential preventive role of metformin in breast cancer survivors and obese patients.	N=2<br>2 randomised controlled trial<br>Publication range: 2020	N=205 endometrial cancer<br>Mean age: 44 years<br>Cancer type: primary diagnosis, recurrent<br>Treatment: adjuvant chemotherapy, fertility-sparing treatment<br>Menopausal status: post-menopausal (n=32)	Abdominal pain<br>Gravidity<br>Parity<br>Treatment-related toxicities	Pregnancy rates<br>Live birth rates	Cochrane Risk of Bias tool: low risk of bias
**Mental Health: ANXIETY**						
**Author (year)**	**Study aim**	**Characteristics of primary studies[a]**	**Characteristics of participants**	**Health-related outcomes reported[#]**	**Measurement instruments[a]**	**Quality appraisal as reported by the review**

Alanazi et al (2021)[23]<br><br>Systematic review	Describe the sleep patterns among uterine cancer survivors and verify psychological and physical factors affecting their general quality of life.	N=10<br>4 randomised controlled trial<br>2 prospective<br>1 retrospective<br>2 cross-sectional<br>1 non-randomised controlled trial<br>Publication range: 2015-2019	N=1535 endometrial cancer<br>Cancer type: stage I<br>Treatment: surgery only, radiotherapy, mixed	Fatigue<br>Obesity<br>Pain<br>Sexual function<br>Sleep[#]<br>Quality of life[#]<br>Anxiety<br>Depression	Actigraphy wristwatch + sleep log<br>BMI<br>BSI-18<br>EORTC QLQ-C30<br>FACT-En<br>PSQI<br>PSS<br>QLACS<br>QOL-CS<br>SF-36	Melnyk's evidence pyramid
Shisler et al (2018)[6]<br><br>Systematic review	Summarize the existing literature related to patient reported outcomes among endometrial cancer survivors and highlight gaps in the literature that should be addressed in future research.	N=27<br>19 cross sectional<br>8 longitudinal<br>Publication range: 2005-2017	N=4315<br>Cancer type: stage I-III<br>Treatment: surgery only, adjuvant chemotherapy, radiotherapy, mixed	Fatigue<br>Pain<br>Sexual function<br>Sleep<br>Quality of life<br>Anxiety<br>Depression<br>Distress/stress	BDI<br>BFI<br>BPI<br>BSI-18<br>EORTC-QLQ-C30<br>FACIT-F<br>FAS<br>IDAS<br>PSQI<br>QLACS<br>SF-36<br>SIGH-AD	NR
**Mental Health: DEPRESSION**						
**Author (year)**	**Study aim**	**Characteristics of primary studies[a]**	**Characteristics of participants**	**Health-related outcomes reported[#]**	**Measurement instruments[a]**	**Quality appraisal as reported by the review**
Alanazi et al (2021)[23]<br><br>Systematic review	Describe the sleep patterns among uterine cancer survivors and verify psychological and physical factors affecting their general quality of life.	N=10<br>4 randomised controlled trial<br>2 prospective<br>1 retrospective<br>2 cross-sectional	N=1535 endometrial cancer<br>Cancer type: stage I<br>Treatment: surgery only, radiotherapy, mixed	Fatigue<br>Obesity<br>Pain<br>Sexual function<br>Sleep[#]<br>Quality of life[#]<br>Anxiety	Actigraphy wristwatch + sleep log<br>BMI<br>BSI-18<br>EORTC QLQ-C30<br>FACT-En	Melnyk's evidence pyramid

		1 non-randomised controlled trial<br>Publication range: 2015-2019		Depression	PSQI<br>PSS<br>QLACS<br>QOL-CS<br>SF-36	
Shisler et al (2018)[6]<br><br>Systematic review	Summarize the existing literature related to patient reported outcomes among endometrial cancer survivors and highlight gaps in the literature that should be addressed in future research.	N=27<br>19 cross sectional<br>8 longitudinal<br>Publication range: 2005-2017	N=4315<br>Cancer type: stage I-III<br>Treatment: surgery only, adjuvant chemotherapy, radiotherapy, mixed	Fatigue<br>Pain<br>Sexual function<br>Sleep<br>Quality of life<br>Anxiety<br>Depression<br>Distress/stress	BDI<br>BFI<br>BPI<br>BSI-18<br>EORTC-QLQ-C30<br>FACIT-F<br>FAS<br>IDAS<br>PSQI<br>QLACS<br>SF-36<br>SIGH-AD	NR
**Mental Health: DISTRESS/STRESS**						
**Author (year)**	**Study aim**	**Characteristics of primary studies[a]**	**Characteristics of participants**	**Health-related outcomes reported[#]**	**Measurement instruments[a]**	**Quality appraisal as reported by the review**
Shisler et al (2018)[6]<br><br>Systematic review	Summarize the existing literature related to patient reported outcomes among endometrial cancer survivors and highlight gaps in the literature that should be addressed in future research.	N=27<br>19 cross sectional<br>8 longitudinal<br>Publication range: 2005-2017	N=4315<br>Cancer type: stage I-III<br>Treatment: surgery only, adjuvant chemotherapy, radiotherapy, mixed	Fatigue<br>Pain<br>Sexual function<br>Sleep<br>Quality of life<br>Anxiety<br>Depression<br>Distress/stress	BDI<br>BFI<br>BPI<br>BSI-18<br>EORTC-QLQ-C30<br>FACIT-F<br>FAS<br>IDAS<br>PSQI<br>QLACS<br>SF-36<br>SIGH-AD	NR

Footnotes:

[a] Only includes details relevant to a health outcome among EC participants.

[#] Denotes primary outcome/s of the review.

Abbreviations:

ACOG: American College of Obstetrics and Gynecologists; AHRQ: Agency for Healthcare Research and Quality; APFQ: Australian Pelvic Floor Questionnaire; BDI: Beck Depression Inventory; BFI: Brief fatigue inventory; BMI: Body Mass Index; BPI: Brief Pain Inventory; BSI-18: Brief Symptom Inventory-18; CALGB: Cancer and Leukemia Group-B; CASP: Critical Appraisal Skills Programme; CaSUN: Cancer Survivors' Unmet Needs Measure; COSMIN: Consensus-based Standards for the Selection of Health Measurement Instruments; CTCAE: Common Terminology Criteria for Adverse Events; EORTC: European Organization for Research and Treatment of Cancer; EORTC-QLQ: European Organisation for Research and Treatment Quality of Life Questionnaire; EORTC-QLQ-C30: European Organisation for Research and Treatment Core Quality of Life Questionnaire – Core 30; EORTC-QLQ-CX24 European Organisation for Research and Treatment Quality of Life Questionnaire Cervical Cancer Module; EORTC-QLQ-EN24: European Organisation for Research and Treatment Quality of Life Questionnaire Endometrial cancer module; EQ-5D: EuroQol-5 Dimension; FACIT: Functional Assessment of Chronic Illness Therapy; FACIT-F: Functional Assessment of Chronic Illness Therapy – Fatigue; FACT-En: Functional Assessment of Cancer Therapy- Endometrial; FACT-G: Functional Assessment of Cancer Therapy- General; FACT-GOG-Ntx: Functional Assessment of Cancer Therapy-Gynecologic Oncology Group-Neurotoxicity symptom scale; FAS: Fatigue Assessment Scale; FSD: female sexual dysfunction; FSFI: Female Sexual Function Index; GCLQ-K: Gynecologic Cancer Lymphedema Questionnaire (Korean version); GLQ: General Lifestyle Questionnaire; GSCQ: Greek Symptom Control Questionnaire; GUPI: Genito-Urinary Pain Index; HADS: Hospital Anxiety and Depression Scale; ICIQ-FLUTS: International Consultation on Incontinence Modular Questionnaire on Female Lower Urinary Tract Symptoms; IDAS: Inventory of Depression and Anxiety Symptoms; IIQ-7: Incontinence Impact Questionnaire-7; IIQ-SF: Incontinence Impact Questionnaire- Short Form; IOC: Impact of Cancer questionnaire; ISI: Insomnia Severity Index; JBI: Joanna Briggs Institute; LENT-SOMA: Late Effects Normal Tissues-Subjective, Objective, Management, Analytic; MINORS: Methodological Index for Non-randomised Studies; MOOSE: Meta-analysis of Observational Studies in Epidemiology; MRI: Medical Resonance Imaging; NOS: Newcastle-Ottawa Scale; NR: Not reported; PEDro: Physiotherapy Evidence Database; PFDI: Pelvic Floor Dysfunction Inventory; PGI-I: Patient Global Impression of Improvement; PISQ-12: Pelvic Organ Prolapse/Urinary Incontinence Sexual Questionnaire; PISQ-IR: Pelvic Organ Prolapse/Urinary Incontinence Sexual Questionnaire- IUGA Revised; PROMIS: Patient Reported Outcome Measurement System; PSQI: Pittsburgh Sleep Quality Index; PSS: Perceived Stress Scale; QLACS: Quality of Life in Adult Cancer Survivors; QOL-CS: Quality of Life-Cancer Survivors; QoR-40: Quality of Recovery-40 Scale; QUID: Questionnaire for Urinary Incontinence Diagnosis; RAND-36: RAND 36-item Health Survey; RI10: Recovery Index-10; ROBANS: Risk of Bias Assessment Tool for Non-randomized Studies; ROBINS-I: Risk Of Bias In Non-randomised Studies of Interventions; RSC: Rotterdam Symptom Checklist; RTOG: Radiation Therapy Oncology Group; SABIS-G: sexual adjustment and body image scale-gynecologic cancer; SAQ: Sexual Activity Questionnaire; SF-12: 12-item Short Form Health Survey; SF-36: 36-item Short Form Health Survey; SHF: Sexual History Form; SIGH-AD: Structured Interview Guide for the Hamilton Anxiety/Depression Scales; SKQ: Sexual knowledge questionnaire; SSFS: Short Sexual Functioning Scale; SSI: Sandvik Severity Index; SSPQ: Specific Sexual Problems Questionnaire; SVQ: The Sexual function-Vaginal changes Questionnaire; UDI-SF: Urinary Distress Inventory-Short Form; VAS: Visual Analogue Scale; WHOQOL-BREF: World Health Organization's Quality of Life instrument-abbreviated version.
